# Systematic analyses uncover plasma proteins linked to incident cardiovascular diseases

**DOI:** 10.1093/procel/pwaf072

**Published:** 2025-08-06

**Authors:** Yi-Lin Chen, Ji-Jing Wang, Jia You, Ji-Yun Cheng, Ze-Yu Li, Yi-Jun Ge, Bing-Ran Yao, Xiao-Yu He, Yu Guo, Yi Zhang, Shi-Dong Chen, Liu Yang, Xin-Rui Wu, Bang-Sheng Wu, Ya-Ru Zhang, Mei Cui, Qiang Dong, Jian-Feng Feng, Mei Tian, Wei Cheng, Jin-Tai Yu

**Affiliations:** Department of Neurology and National Center for Neurological Disorders, Huashan Hospital, State Key Laboratory of Medical Neurobiology and MOE Frontiers Center for Brain Science, Fudan University, Shanghai 200433, China; Huashan Hospital & Human Phenome Institute, Fudan University, Shanghai 200433, China; Department of Neurology, Brigham and Women’s Hospital, Mass General Brigham, Boston, MA 02115, United States; Harvard Medical School, Boston, MA 02115, United States; Institute of Science and Technology for Brain-Inspired Intelligence (ISTBI), Fudan University, Shanghai 200433, China; Department of Neurology and National Center for Neurological Disorders, Huashan Hospital, State Key Laboratory of Medical Neurobiology and MOE Frontiers Center for Brain Science, Fudan University, Shanghai 200433, China; Institute of Science and Technology for Brain-Inspired Intelligence (ISTBI), Fudan University, Shanghai 200433, China; Department of Neurology and National Center for Neurological Disorders, Huashan Hospital, State Key Laboratory of Medical Neurobiology and MOE Frontiers Center for Brain Science, Fudan University, Shanghai 200433, China; Department of Neurology and National Center for Neurological Disorders, Huashan Hospital, State Key Laboratory of Medical Neurobiology and MOE Frontiers Center for Brain Science, Fudan University, Shanghai 200433, China; Department of Neurology and National Center for Neurological Disorders, Huashan Hospital, State Key Laboratory of Medical Neurobiology and MOE Frontiers Center for Brain Science, Fudan University, Shanghai 200433, China; Department of Neurology and National Center for Neurological Disorders, Huashan Hospital, State Key Laboratory of Medical Neurobiology and MOE Frontiers Center for Brain Science, Fudan University, Shanghai 200433, China; Department of Neurology and National Center for Neurological Disorders, Huashan Hospital, State Key Laboratory of Medical Neurobiology and MOE Frontiers Center for Brain Science, Fudan University, Shanghai 200433, China; Department of Neurology and National Center for Neurological Disorders, Huashan Hospital, State Key Laboratory of Medical Neurobiology and MOE Frontiers Center for Brain Science, Fudan University, Shanghai 200433, China; Department of Neurology and National Center for Neurological Disorders, Huashan Hospital, State Key Laboratory of Medical Neurobiology and MOE Frontiers Center for Brain Science, Fudan University, Shanghai 200433, China; Department of Neurology and National Center for Neurological Disorders, Huashan Hospital, State Key Laboratory of Medical Neurobiology and MOE Frontiers Center for Brain Science, Fudan University, Shanghai 200433, China; Department of Neurology and National Center for Neurological Disorders, Huashan Hospital, State Key Laboratory of Medical Neurobiology and MOE Frontiers Center for Brain Science, Fudan University, Shanghai 200433, China; Department of Neurology and National Center for Neurological Disorders, Huashan Hospital, State Key Laboratory of Medical Neurobiology and MOE Frontiers Center for Brain Science, Fudan University, Shanghai 200433, China; Department of Neurology and National Center for Neurological Disorders, Huashan Hospital, State Key Laboratory of Medical Neurobiology and MOE Frontiers Center for Brain Science, Fudan University, Shanghai 200433, China; Department of Neurology and National Center for Neurological Disorders, Huashan Hospital, State Key Laboratory of Medical Neurobiology and MOE Frontiers Center for Brain Science, Fudan University, Shanghai 200433, China; Institute of Science and Technology for Brain-Inspired Intelligence (ISTBI), Fudan University, Shanghai 200433, China; Key Laboratory of Computational Neuroscience and Brain-Inspired Intelligence, Ministry of Education, Fudan University, Shanghai 200433, China; Huashan Hospital & Human Phenome Institute, Fudan University, Shanghai 200433, China; Department of Nuclear Medicine/PET Center, Huashan Hospital, Fudan University, Shanghai 200433, China; Department of Neurology and National Center for Neurological Disorders, Huashan Hospital, State Key Laboratory of Medical Neurobiology and MOE Frontiers Center for Brain Science, Fudan University, Shanghai 200433, China; Institute of Science and Technology for Brain-Inspired Intelligence (ISTBI), Fudan University, Shanghai 200433, China; Key Laboratory of Computational Neuroscience and Brain-Inspired Intelligence, Ministry of Education, Fudan University, Shanghai 200433, China; Department of Neurology and National Center for Neurological Disorders, Huashan Hospital, State Key Laboratory of Medical Neurobiology and MOE Frontiers Center for Brain Science, Fudan University, Shanghai 200433, China

**Keywords:** cardiovascular disease, plasma proteins, prediction model, biomarker, longitudinal study

## Abstract

Cardiovascular disease (CVD) research is hindered by limited comprehensive analyses of plasma proteome across disease subtypes. Here, we systematically investigated the associations between plasma proteins and cardiovascular outcomes in 53,026 UK Biobank participants over a 14-year follow-up. Association analyses identified 3,089 significant associations involving 892 unique protein analytes across 13 CVD outcomes. The most notable associations included NT-proBNP for atrial fibrillation (*P* = 6.31 × 10^−313^), followed by NPPB (*P* = 1.03 × 10^−164^) and GDF15 for heart failure (*P* = 1.21 × 10^−166^). Among 445 unique proteins significantly linked to 18 cardiovascular metrics, LEP (RVEDV: β = −9.03, *P* = 2.76 × 10^−51^) and FABP4 (RVEDV: β = −10.18, *P* = 2.42 × 10^−32^) emerged as the strongest correlates of cardiac structure and function. Our integrated prediction model performed excellently across the majority of CVD outcomes, achieving an AUC of 0.86 for abdominal aneurysm. Two-sample Mendelian randomization analysis revealed 225 proteins causally linked to CVDs, with LPA showing the strongest coronary artery disease association (OR = 1.13 [1.10–1.17], *P* = 2.38 × 10^−15^), many of which are targets of existing drugs, suggesting repurposing opportunities. Mediation analysis revealed broad-spectrum mediators (e.g., IGFBP4 and GDF15, each influencing 9 cardiovascular outcomes) and outcome-specific protein mediators, with modifiable risk factors such as smoking and BMI predominantly mediating protein-CVD associations.This comprehensive longitudinal study provides unprecedented insights into plasma proteome influences on cardiovascular health interactions, offering novel perspectives for CVD diagnosis, prediction, and prevention.

## Introduction

Cardiovascular diseases (CVDs) involve a broad group of pathological lesions in the blood vessels and heart, constituting approximately 30% of global deaths and imposing a substantial economic burden (Cannon, 2013; Tsao et al., 2022). Various conditions, characterized by structural or functional changes in the heart or blood vessels, contribute to a diverse array of CVD subtypes, including cerebrovascular diseases, thromboembolic diseases, and others. Heterogeneity exists at multiple levels, from genotype to clinical phenotype in CVDs, especially considering complex molecular interactions, interrelated disease features, and variable clinical trajectories. Unfortunately, current diagnostic tools, such as cardiovascular magnetic resonance (CMR), tissue biopsies, and implantable assist devices, face limitations due to technical constraints, invasiveness, or upfront costs (Lam et al., 2023; Parwani et al., 2023; Ranard et al., 2023). Moreover, current treatments such as thrombolysis and surgery present a significant residual risk with a poor prognosis (Heymans et al., 2023; Liu et al., 2023; Tfelt-Hansen et al., 2023), and several drugs for CVD entail serious complications (Lam et al., 2023; Zhang et al., 2023). This challenging scenario highlights the necessity of identifying the heightened risk early before the onset of cardiovascular events, especially incident CVDs. Early detection enables healthcare professionals to anticipate pathophysiological responses and enhance interventional opportunities.

Blood tests, known for their simplicity and lower cost, have gained attention as a valuable alternative in clinical applications of diseases with heterogeneity (Macklin et al., 2020; Teunissen et al., 2022). Particularly, plasma proteins, established as biomarkers for CVD occurrence and prognosis (Blaz et al., 2023; Itzhar et al., 2023), have been explored in their connection to cardiometabolism (Liu and Chen, 2023), cardiovascular drugs’ impact (Lu et al., 2023), and CVD risk factors (Duan et al., 2023), providing valuable insights into CVD monitoring and screening. A recent retrospective study even showed a protein risk score’s modestly better discriminatory capacity for atherosclerotic CVD compared to polygenic risk score (Helgason et al., 2023). However, potential reverse causations compromise the reliability of these correlations, and relatively small sample sizes in cross-sectional studies further limit robustness. To address these limitations and establish potential causal relationships, Mendelian randomization (MR) presents a valuable approach by utilizing genetic variants as instrumental variables, thereby minimizing confounding and reverse causation issues. This method is particularly relevant for investigating plasma proteins, as genetic determinants of protein levels can serve as natural proxies for causal inference. Moreover, a critical gap exists, with no prior studies directly comparing different subtypes of CVDs or examining the relationship of plasma proteome with cardiac structures and functions. This lack of research hampers the ability to discern variations in biomarker performance across a spectrum of cardiovascular conditions. In addition, given that both genetic and cardiovascular health metrics contribute to CVD development, unraveling the elusive mechanisms underlying these associations could establish foundations for more effective therapeutic interventions. Therefore, a consistent and systematic longitudinal study with a larger cohort involving a broad range of incident CVDs is needed to reveal the role of plasma proteins in the disease pre-pathogenesis and progression, paving the way for more effective intervention and treatment strategies for this lethal disease.

**Figure 1.**
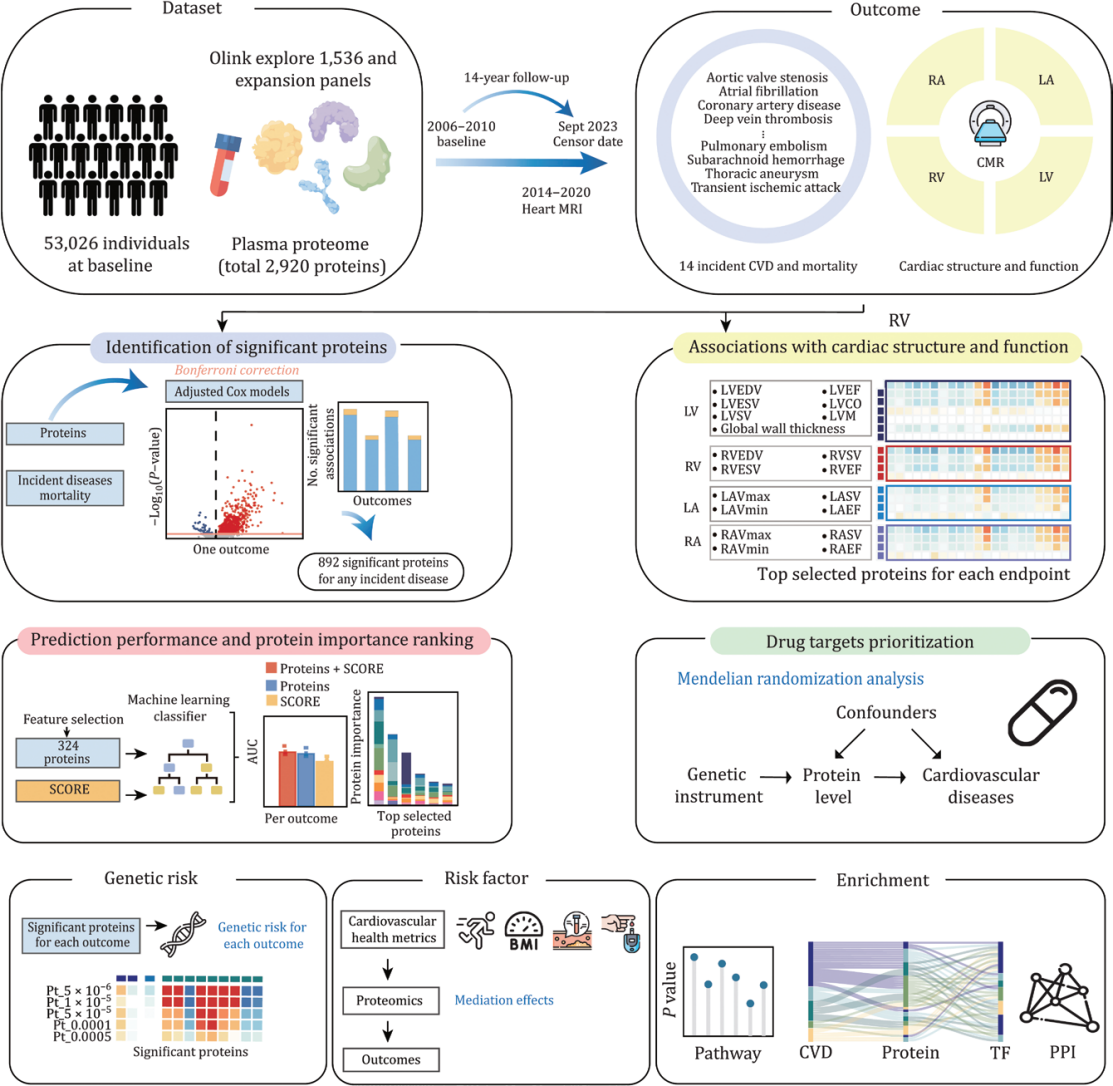
Graphical abstract. In our comprehensive study, we meticulously examined 2,920 plasma proteins measured longitudinally in a large cohort of 53,026 individuals from the baseline period of 2006–2010 to the censor date in September 2023, providing a robust median 14-year follow-up. Our investigation encompassed a spectrum of outcomes, including 14 incident CVDs, mortality, and various cardiac structural and functional parameters. Utilizing Cox models adjusted for demographic and lifestyle factors, we identified 892 proteins significantly associated with the 14 CVD outcomes and mortality after stringent Bonferroni correction. Additionally, we delved into the associations between the plasma proteome and 19 Cardiovascular Magnetic Resonance metrics using linear regression models. Employing a machine-learning-based prediction model trained on 257 pre-selected proteins and three sets of predictors (Protein, SCORE2, and Protein + SCORE2), we demonstrated the enhanced performance of plasma proteins in differentiating individuals developing CVDs from their healthy counterparts, thereby highlighting their supplementary contribution alongside established risk scales. Our investigation extended to a two-sample MR analysis, leveraging GWAS to assess causal associations of the plasma proteome with CVDs. Furthermore, we evaluated the genetic risk for each CVD outcome through polygenic risk scoring. Formal mediation analysis was employed to scrutinize dependencies among the plasma proteome, specific risk factors, and incident CVDs. Lastly, we conducted functional enrichment to map related pathways and construct a protein–protein interaction network, contributing to a holistic understanding of the complex interplay between the plasma proteome and cardiovascular health. The icons used in this figure were sourced from Figdraw and Flaticon.

In this study, via Cox proportional hazard models we examined the association between levels of 2,920 baseline plasma proteins and 14 CVD outcomes and mortality in 53,026 participants within the prospective UK Biobank cohort (Fig. 1). Using linear regression, we extensively investigated the relationship between levels of identified proteins and CMR metrics. A prediction model was employed to evaluate the performance of selected proteins in predicting incident CVDs. Subsequently, the CVD-related proteins were connected to genetic indicators through Mendelian randomization to establish causal relationships, and we further explored their potential as therapeutic targets for CVD prevention and treatment. Finally, the risk factors mediated by plasma proteins were illustrated, and functional enrichment analyses were undertaken to map biological pathways and protein-protein interaction networks. Altogether, the current study facilitates a holistic understanding of the complex interplay between the plasma proteome and cardiovascular health, thereby opening new avenues for predicting and preventing CVDs through innovative research and targeted interventions.

## Results

### Participants

In this study, a total sample of 53,026 participants with an average age of 53.8 years (standard deviation [SD] 8.1) was included, comprising 55% women and 94% white individuals (Fig. 1). After a median follow-up period of 14.5 years (interquartile range [IQR] 13.8–15.3), 1,876 participants experienced mortality. In the subgroup comprising 46,818 participants initially free of any type of CVDs, 9,096 participants were diagnosed with incident CVDs. Table S1 provides an overview of the baseline characteristics of participants.

### Cox models correlated plasma proteome with various types of incident CVDs

**Figure 2.**
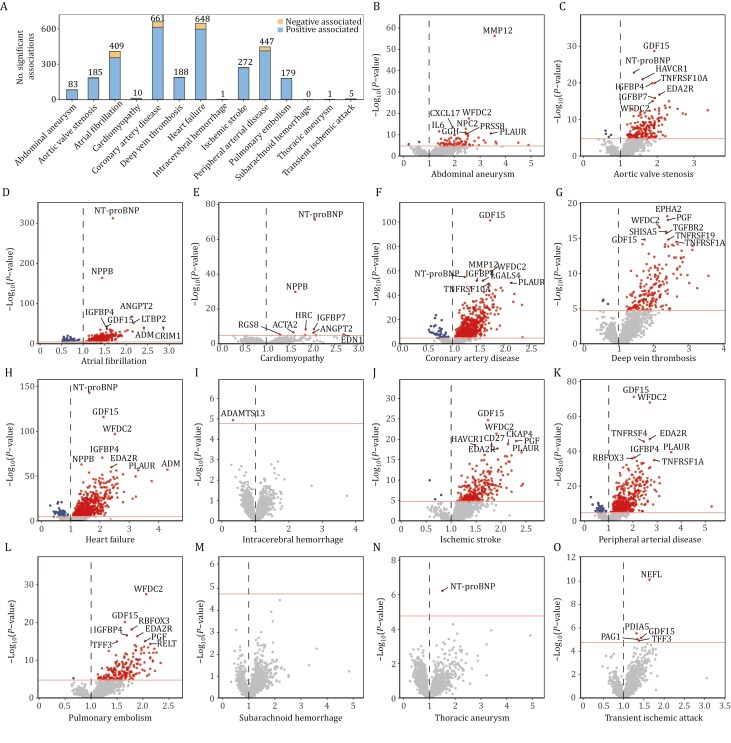
Cox proportional hazard model correlates plasma proteome with CVD. (A) Numbers of significant associations between protein levels and 14 incident CVD outcomes. (B‒O) Volcano plots presenting the association of protein expressions with each outcome. The x-axis shows the HR, indicating the strength and direction of the association between protein levels and incident. The y-axis indicates the −log_10_ of the *P*-value for each association. Proteins with a *P*-value below the threshold determined by the Bonferroni correction (*P* < 0.05/2,920), are depicted above the black horizontal line. The red dots represent risk proteins, suggesting that they are significantly associated with an enhanced risk of incident CVD, while the blue dots represent protective proteins, indicating that they are significantly associated with a reduced risk of incident CVD. The top 8 proteins with highest −log_10_(*P*-value) for each outcome are marked with their names. The Cox proportional hazard models were adjusted for age, sex, ethnicity, TDI, blood collection season, time between protein measurement and blood sampling (in days), participant-reported fasting time, systolic blood pressure, BMI, smoking status, and alcohol intake.

Using Cox proportional hazard models, we explored the relationship between plasma protein levels and 14 incident CVD outcomes including cerebrovascular diseases (ischemic stroke, intracerebral hemorrhage, subarachnoid hemorrhage, and transient ischemic attack), and other CVDs (pulmonary embolism, deep vein thrombosis atrial fibrillation, coronary artery disease, cardiomyopathy, heart failure, and peripheral artery disease; Fig. S1 and Table S2). Meanwhile, we adjusted for age, sex, ethnicity, townsend deprivation index (TDI), blood collection season, time between protein measurement and blood sampling (in days), participant-reported fasting time, systolic blood pressure (SBP), body mass index (BMI), smoking status, and alcohol intake. After Bonferroni correction (*P* < 0.05/2,920), there were 3,089 associations between 892 unique protein analytes and 13 outcomes. Notably, coronary artery disease and heart failure have the most association numbers (> 600), while no significant association was found for subarachnoid hemorrhage (Fig. 2A). Most of the significant correlations found were positive (89%). Volcano plots illustrating protein expression for the onset of each outcome are present in Fig. 2B‒O. All associations, after Bonferroni correction, were found to be positive for cardiomyopathy, transient ischemic attack, and thoracic aneurysm. Intriguingly, only N-terminal prohormone of brain natriuretic peptide (NT-proBNP) had a positive correlation with thoracic aneurysm, while only a disintegrin and metalloproteinase with thrombospondin motifs 13 (ADAMTS13) were negatively associated with intracerebral hemorrhage. We also investigated the association of plasma proteome with mortality in different CVD outcomes, and 783 proteins were significantly associated with mortality in 12 outcomes, except for thoracic aneurysm and transient ischemic attack (Fig. S2 and Table S3). As mortality may act as a competing risk, particularly in high-fatality subtypes, we conducted supplementary Fine-Gray models for selected outcomes, which showed consistent results (Table S4).

Multimorbidity profiling identified 262 proteins associated with 5 or more incident CVDs (Fig. S3A), while 61 proteins were significantly correlated with mortality in 5 or more CVD outcomes (Fig. S3B). Among them, tumor necrosis factor receptor superfamily member 27 (EDA2R), growth differentiation factor 15 (GDF15), and NT-proBNP had the largest number of associations—with 10 incident CVD outcomes. The most significant associations were observed for NT-proBNP with atrial fibrillation (HR = 1.69, 95% CI: 1.65‒1.74, *P* = 6.31 × 10^−313^), followed by natriuretic peptide B (NPPB) with atrial fibrillation (HR = 1.44 [1.41‒1.48], *P* = 1.03 × 10^−164^), NT-proBNP with heart failure (HR = 1.64 [1.58‒1.70], *P* = 8.97 × 10^−144^), and GDF15 with heart failure and CAD (HR = 2.12 [1.99‒2.26], *P* = 1.21 × 10^−166^; HR = 1.73 [1.64‒1.81], *P* = 4.62 × 10^−102^). Additionally, Macrophage metalloelastase (MMP12), EDA2R, latent transforming growth factor beta binding protein 2 (LTBP2), insulin-like growth factor binding protein 7 (IGFBP7), WAP four-disulfide core domain 2 (WFDC2), and placental growth factor (PGF) were also among the top 20 proteins strongly associated with incident CVD (Table S2). Specifically, MMP12 and WFDC2 exhibited the strongest associations with abdominal aneurysm and were ranked among the top three for coronary artery disease. For associations with deep vein thrombosis and ischemic stroke, WFDC2 and PGF were among the top three. WFDC2 also showed the strongest correlation with heart failure and pulmonary embolism. Besides, NT-proBNP was most strongly associated with cardiomyopathy and heart failure, and had the second highest association with aortic valve stenosis. WFDC2 and EDA2R were among the top three for peripheral arterial disease, while LTBP2 was ranked third for atrial fibrillation.

When conducting sensitivity analyses to examine the impact of age (≥ 60) and sex on Cox proportional hazard models, we observed varying effect sizes and correlation coefficients across different CVD outcomes (Fig. S4; Tables S5 and S6). The associations remained robust in sensitivity analyses with additional adjustment for creatinine, cystatin-C, LDL-cholesterol, HDL-cholesterol, C-reactive protein, hemoglobin, and HbA1c (Table S7).

### Linear regression linked plasma proteins to CMR metrics

**Figure 3.**
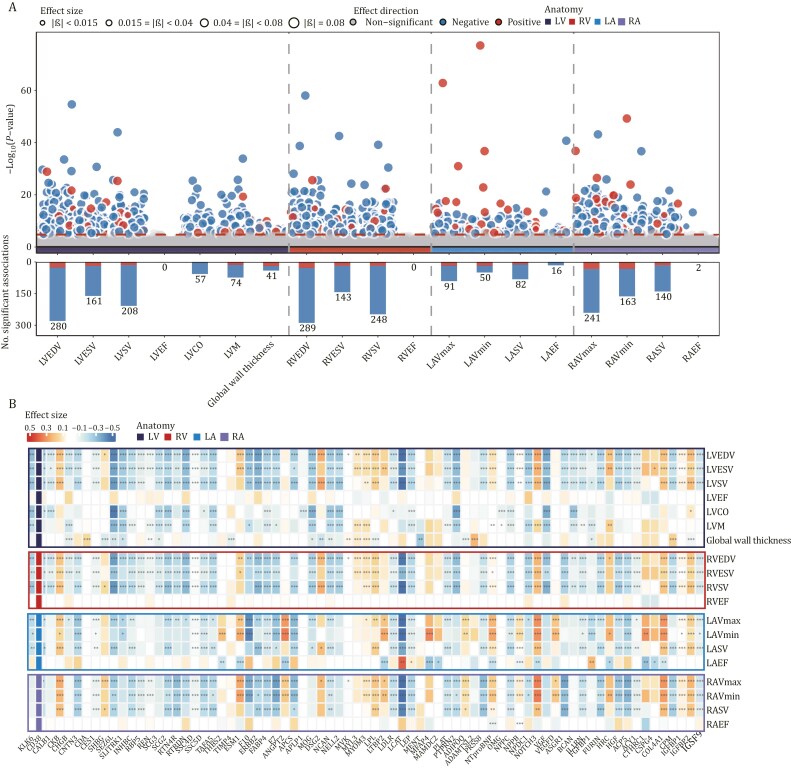
Linear regression links plasma proteins to cardiovascular magnetic resonance (CMR) metrics. (A) Manhattan plot displays the associations between plasma proteins and 19 CMR metrics. The significance threshold was adjusted to account for multiple comparisons, and unadjusted –log_10_(*P*-value) values are reported. Each circle represents a single protein-CMR association, with size indicating the regression coefficient The horizontal dashed line indicates that the Bonferroni-corrected threshold of –log_10_(*P*-value) is 4.76, above which the associations are considered significant. Dashed vertical lines separate five different phenotype categories: LV, RV, LA, and RA. (B) The heat map illustrates the association of each CMR metric with its most significantly correlated (top-15) plasma proteins. The color gradient from blue to red suggests the regression coefficient β. *, *P* < 0.05/2,920; **, *P* < 0.01/2,920; ***, *P* < 0.001/2,920. Multiple linear regression models were adjusted for age, sex, ethnicity, TDI, blood collection season, time between protein measurement and blood sampling (in days), participant-reported fasting time, systolic blood pressure, BMI, smoking status, and alcohol intake.

We investigated associations between plasma proteome and 19 key CMR metrics, capturing cardiac dimensions (mass, volume, or thickness) and their dynamic changes (ejection fraction) throughout the cardiac cycle. These metrics encompassed four anatomical structures: left ventricle (LVEDV, LVESV, LVSV, LVEF, LVCO, LVM, and global wall thickness), right ventricle (RVEDV, RVESV, RVSV, RVEF), left atrium (LAV max, LAV min, LASV, LAEF), and right atrium (RAV max, RAV min, RAEF, RASV). After executing the analysis pipeline and conducting quality control, these imaging phenotypes were available for 4,287 participants. Table S8 provides the summary statistics for the cardiac structures and functions of participants. In total, 441 unique proteins were significantly associated with 18 cardiovascular phenotypes after Bonferroni correction (*P* < 1.71 × 10^−5^, 0.05/2,920; Fig. 3A, Table S9). Notably, RVEDV (*n* = 289) and LVEDV (*n* = 280) had the largest number of associated proteins, while no significant association was discovered for RVEF and LVEF. This differential pattern was consistent across chambers, where volumetric measurements (EDV, ESV, SV) generally showed more protein associations than functional parameters (EF). This suggests that circulating proteins may be more strongly associated with cardiac structural remodeling than with functional performance. However, this difference could also reflect the greater susceptibility of EF to short-term physiological influences—such as preload, afterload, or neurohumoral tone—compared to volumetric traits, which may more reliably capture stable anatomical features. The biological basis for this divergence remains unclear, and the potential impact of hemodynamic confounders and measurement variability warrants further investigation.

Among the 441 proteins with significant associations, we identified a core group extensively connected with cardiac parameters. A heatmap depicting the regression coefficients and significance levels for the top 15 proteins associated with each CMR metric (75 proteins in total) is presented in Fig. 3B. Leptin (LEP) and NT-proBNP showed the most extensive connections (each associated with 15 cardiac parameters), followed by fatty acid-binding protein, adipocyte (FABP4), insulin-like growth factor binding protein 2 (IGFBP2), neurocan core protein (NCAN), neurogenic locus notch homolog protein 3 (NOTCH3), C-type natriuretic peptide (NPPC), and seizure 6-like protein (SEZ6L) (each linked to 14 parameters). These proteins exhibited distinct chamber-specific patterns. NT-proBNP demonstrated the strongest associations with atrial volumes (LAV min: β = 0.260, *P* = 6.29 × 10^−78^; LAV max: β = 0.229, *P* = 1.52 × 10^−63^), while maintaining significant but weaker associations with ventricular parameters. In contrast, LEP exhibited consistent negative associations across chambers, with strongest effects on ventricular volumes (RVEDV: β = −0.243, *P* = 9.70 × 10^−59^; LVEDV: β = −0.249, *P* = 2.39 × 10^−55^). These differential patterns suggest distinct molecular mechanisms regulating atrial versus ventricular regulation. Notably, we observed symmetrical effects of several proteins between left and right chambers, highlighting their potential roles in overall cardiac size regulation. For instance, FABP4 showed comparable negative correlations with both RVEDV (β = −0.284, *P* = 1.94 × 10^−39^) and LVEDV (β = −0.278, *P* = 3.10 × 10^−34^). Similarly, IGFBP2 demonstrated consistently positive associations across multiple cardiac chambers, with comparable effects on ventricular measures (LVEDV: β = 0.205, *P* = 1.49 × 10^−29^; LVSV: β = 0.209, *P* = 4.96 × 10^−26^; RVEDV: β = 0.183, *P* = 2.57 × 10^−26^; RVSV: β = 0.191, *P* = 5.09 × 10^−23^), as well as atrial volumes (RAV max: β = 0.228, *P* = 3.64 × 10^−27^; LAV max: β = 0.163, *P* = 8.01 × 10^−14^).

The regulatory network revealed intriguing bidirectional effects. FABP4 and NCAN, despite their widespread correlations, showed predominantly negative associations with cardiac volumes (e.g., FABP4-RVEDV: β = −0.284, *P* = 1.94 × 10^−39^; NCAN-LVEDV: β = −0.280, *P* = 2.81 × 10^−22^). In contrast, IGFBP2 exhibited opposing positive correlations (LVEDV: β = 0.205, *P* = 1.49 × 10^−29^). Similarly, SEZ6L, while broadly connected, displayed its strongest negative association with cardiac output (LVCO: β = −0.477, *P* = 4.04 × 10^−26^), whereas NOTCH3 showed its strongest effect as positive correlations with atrial volumes (RAV min: β = 0.343, *P* = 2.50 × 10^−20^). Furthermore, we identified distinct patterns in protein associations across cardiac chambers. Atrial volume measures showed stronger associations with natriuretic peptides (NT-proBNP, NPPB), while ventricular volumes demonstrated stronger correlations with metabolic regulators (LEP, FABP4).

Of note, phosphatidylcholine-sterol acyltransferase (LCAT) emerged as a protein with striking chamber-specific effects and clear directional patterns. It showed the strongest negative associations with chamber volumes across all four cardiac chambers, particularly with atrial measurements (RAV max: β = −0.603, *P* = 1.30 × 10^−16^; LAV max: β = −0.556, *P* = 5.56 × 10^−13^) and ventricular volumes (LVEDV: β = −0.527, *P* = 5.89 × 10^−17^; RVEDV: β = −0.484, *P* = 8.22 × 10^−16^). Intriguingly, while LCAT demonstrated consistent negative correlations with structural parameters (including end-diastolic volumes, end-systolic volumes, and stroke volumes), it showed positive associations with atrial function, most notably with left atrial ejection fraction (LAEF: β = 0.397, *P* = 9.16 × 10^−7^).

### Enhancing CVD prediction with plasma proteins

**Figure 4.**
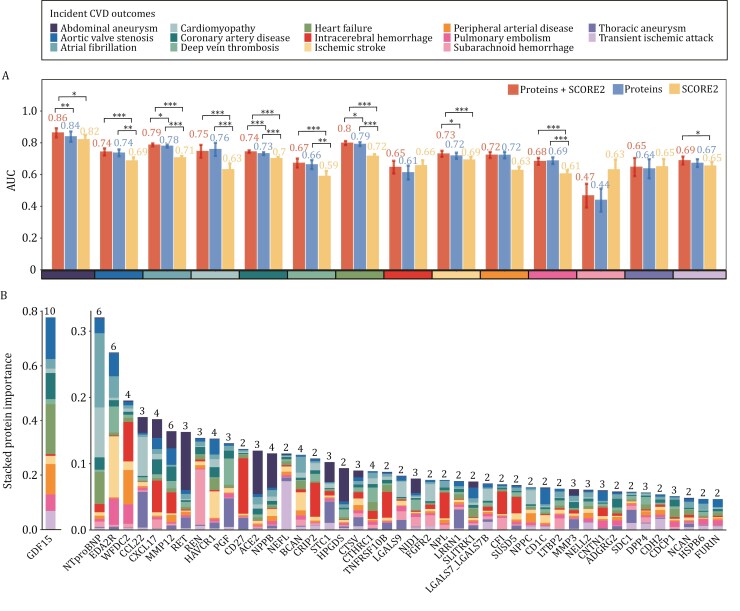
Receiver Operating Characteristic analyses proved the feasibility of predicting incident CVD. (A) Bar plot displaying the AUCs of predictive models established based on three predictor sets: Protein (selected 257 plasma proteins), SCORE2 and Protein + SCORE2. Error bars represent the interquartile range derived by bootstrapping with 1000 iterations. ***, **, and * indicate significant difference tested using DeLong statistics with *P*-values < 0.001, < 0.01, and < 0.05, respectively. (B) Stacked bar chart showing protein importance calculated based on standardized information gain derived from LGBM classifiers. The top-15 proteins for each CVD outcome were selected and ranked based on their overall importance. Numbers on top of the bars indicate the amount of CVDs in which this protein ranked within the top-15 list. Thus we showed proteins that exhibited the most important discriminative value in two or more CVDs.

To evaluate the clinical utility of plasma proteins in cardiovascular disease prediction, we developed and compared three machine learning models: a protein-based model incorporating 257 pre-selected plasma proteins, the established SCORE2 risk scale (SCORE2 working group and ESC Cardiovascular risk collaboration, 2021) (integrating conventional risk factors including age, total cholesterol, high-density lipoprotein [HDL] cholesterol, systolic blood pressure, prevalent diabetes, and smoking status), and and an integrated model combining both protein markers and SCORE2 (Protein + SCORE2) (Table S10 and Fig. 4A). The protein-based model demonstrated significantly superior predictive performance compared to SCORE2 in seven cardiovascular outcomes (*P* < 0.05), with particularly strong improvements in atrial fibrillation (area under the curve [AUC]: 0.779 [0.765‒0.793] vs. 0.707 [0.692‒0.721], *P* < 0.001), cardiomyopathy (AUC: 0.759 [0.697‒0.814] vs. 0.631 [0.575‒0.683], *P* < 0.001), and peripheral arterial disease (AUC: 0.723 [0.692‒0.751] vs. 0.627 [0.596‒0.660], *P* < 0.001). Furthermore, the integration of plasma proteins with SCORE2 yielded significant improvements over SCORE2 alone across eleven cardiovascular outcomes (Proteins + SCORE2 > SCORE2, *P* < 0.05), with particularly strong improvements in abdominal aortic aneurysm (AUC: 0.863 [0.818‒0.904] vs. 0.821 [0.779‒0.856], *P* < 0.05), atrial fibrillation (AUC: 0.787 [0.773‒0.800] vs. 0.707 [0.692‒0.721], *P* < 0.001), and heart failure (AUC: 0.798 [0.778‒0.817] vs. 0.716 [0.696‒0.735], *P* < 0.001).

To further evaluate the clinical utility of protein biomarkers beyond the SCORE2 risk model, we quantified incremental predictive performance using net reclassification improvement (NRI) and integrated discrimination improvement (IDI) for the integrated model (Protein + SCORE2) versus SCORE2 alone. The integrated model demonstrated statistically significant reclassification improvement (NRI > 0, 95% CI excluding zero) for nine endpoints. Substantial enhancements were observed for Cardiomyopathy (NRI = 0.185 [95% CI: 0.072‒0.297]), HF (NRI = 0.133 [95% CI: 0.091‒0.174]) and AF (NRI = 0.133 [95% CI: 0.102‒0.164]). Similarly, significant discrimination improvement (IDI > 0, 95% CI excluding zero) were observed in ten endpoints, most notably in AF (IDI = 0.064 [95% CI: 0.056‒0.072]) and HF (IDI = 0.050 [95% CI: 0.041‒0.059]) (Table S11).

The importance of each protein was calculated based on standardized information gain, which was determined through LGBM classifiers (Table S12). To identify proteins with broad cardiovascular relevance, we selected proteins ranked among the top 15 predictors in multiple (≥ 2) conditions, with their cumulative importance scores shown in Fig. 4B. GDF15 emerged as the strongest predictor across cardiovascular diseases (cumulative score 0.78), appearing for 10 conditions, with highest importance in heart failure, aortic valve stenosis and peripheral arterial disease. NT-proBNP (0.32) and EDA2R (0.27) were included in the top 15 for six conditions each. NT-proBNP showed particular strength in atrial fibrillation and cardiomyopathy, while EDA2R demonstrated the highest predictive value for ischemic stroke and pulmonary embolism. MMP12 (0.15) also ranked among the top predictors in multiple conditions, showing specific importance in peripheral arterial disease and deep vein thrombosis. Among proteins appearing in four conditions, WFDC2 (0.20) showed specific importance in cerebrovascular and peripheral vascular diseases, while C-X-C motif chemokine ligand 17 (CXCL17) (0.17) and hepatitis A virus cellular receptor 1 (HAVCR1) (0.14) were particularly predictive for CAD and heart failure. NPPB and brevican core protein (BCAN) (both appearing in four conditions) demonstrated specific importance in atrial fibrillation and peripheral arterial disease. For disease-specific markers, C-C motif chemokine ligand 22 (CCL22) and proto-oncogene tyrosine-protein kinase receptor Ret (RET) showed notable importance in aneurysm-related conditions, while CD27 molecule (CD27) and neurofilament light polypeptide (NEFL) demonstrated particular predictive value for cerebrovascular events.

### Drugs targeting the CVD-associated protein genes were identified

**Figure 5.**
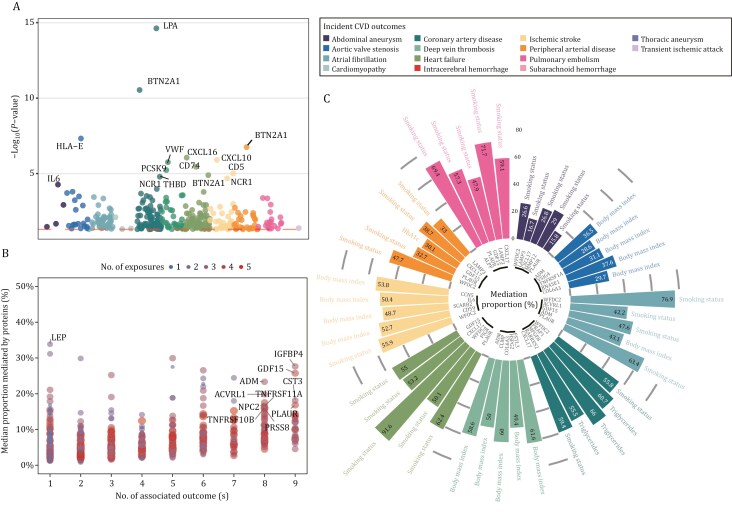
Causal relationship of plasma proteins with incident CVDs and mediation effects of plasma proteins between modifiable factors and CVDs. (A) Manhattan plot showing the statistical significance (−log_10_[*P*-value]) of protein-cardiovascular disease associations derived from inverse variance weighted (IVW) Mendelian randomization analyses. Each point represents a unique protein-outcome association, with different colors indicating distinct cardiovascular outcomes. (B) The scatter plot illustrates the relationship between the number of associated cardiovascular outcomes (x-axis) and the median proportion mediated by proteins (y-axis). Each point represents a unique protein mediator, with point size and color intensity indicating the number of risk factors involved in the mediation pathway. (C) The bar plots indicate the five maximum amounts of mediation proportion for any proteins between risk factors and each CVD. The names of proteins and risk factors were annotated.

To explore the potential causal impact of CVD-associated proteins on the disease onset, a two-sample Mendelian randomization (MR) employing the inverse variance weighted (IVW) method was conducted. Specifically, genome-wide association analysis (GWAS) was performed for each of the 892 CVD-associated proteins, utilizing participants with proteome data to identify protein quantitative trait loci. The results provided evidence for a causal relationship between higher levels of 225 proteins and major CVDs (Fig. 5A and Table S13). Notably, lipoprotein(a) (LPA) showed the strongest causal association with CAD (OR = 1.13, 95% CI: 1.10‒1.17, *P* = 2.38 × 10^−15^). We identified several proteins with pleiotropic effects on multiple CVDs: butyrophilin subfamily 2 member A1 (BTN2A1) demonstrated robust causal effects on CAD (*P* = 2.90 × 10^−11^, OR = 1.11 [1.08‒1.15]), peripheral arterial disease (*P* = 1.76 × 10^−7^, OR = 1.40 [1.24‒1.59]), and heart failure (*P* = 1.27 × 10^−5^, OR = 1.08 [1.05‒1.12]); HLA class I histocompatibility antigen, alpha chain E (HLA-E) showed consistent associations with aortic valve stenosis (*P* = 4.73 × 10^−8^, OR = 1.15 [1.10‒1.22]), deep vein thrombosis (*P* = 2.68 × 10^−4^, OR = 1.14 [1.06‒1.22]), peripheral arterial disease (*P* = 1.84 × 10^−3^, OR = 1.20 [1.07‒1.35]), heart failure (*P* = 1.20 × 10^-3^, OR = 1.06 [1.02‒1.10]), and CAD (*P* = 5.95 × 10^-3^, OR = 1.06 [1.02‒1.10]). Additionally, C-X-C motif chemokine ligand 10 (CXCL10) was causally linked to ischemic stroke (*P* = 1.24 × 10^−6^, OR = 1.62 [1.33‒1.97]), and von Willebrand factor (VWF) to deep vein thrombosis (*P* = 1.75 × 10^−6^, OR = 1.66 [1.35‒2.05]). These findings were robust across multiple sensitivity analyses (MR-Egger, weighted median, and mode). Reverse MR analysis revealed only 23 proteins were influenced by CVDs, suggesting most protein alterations were causal factors rather than disease consequences (Table S14).

To evaluate the therapeutic potential of MR-identified proteins, we performed drug target enrichment analysis using the GREP database (Table S15). This analysis revealed that several causal proteins are already targeted by existing cardiovascular medications, validating our MR findings. Notably, for ischemic heart diseases (I20–I25), we identified multiple established drug targets, including proprotein convertase subtilisin/kexin type 9 (PCSK9) (targeted by evolocumab and alirocumab), coagulation factor X (F10) (targeted by apixaban), and LPA (targeted by ISIS-APO(a)). For diseases of arteries, arterioles, and capillaries (I70–I79), we found significant enrichment of drug targets, with proteins such as F10, F2R thrombin receptor (F2R) (targeted by vorapaxar), and vascular endothelial growth factor A (VEGFA) (targeted by ranibizumab) being actively targeted. The analysis also revealed therapeutic applications in cerebrovascular diseases (I60–I69), where proteins like poly (ADP-ribose) polymerase 1 (PARP1) are targeted by multiple compounds, including nicaraven and hydamtiq. Particularly noteworthy was the enrichment of targets in venous diseases (I80–I89), where numerous F10 inhibitors (including apixaban, betrixaban, and rivaroxaban) and other anticoagulants are currently in use.

### The majority of protein-CVD associations are influenced by modifiable risk factors

To discern the intricate dependencies among the plasma proteome, specific risk factors, and incident CVDs, we employed a formal mediation analysis framework. Triplets were meticulously matched among risk factors, protein levels, and CVD outcomes through the development of Cox models for 14 baseline characteristics chosen for their clinical utility (Methods). Out of a total of 6,665 possible paths representing significant and directionally consistent triangles between risk factor-protein level-CVD status, a substantial majority, comprising 4,757 (71.4%), demonstrated a significant mediation effect (*P* < 0.05/6,665). This finding underscores the intricate relationships between proteins and risk factors in the context of specific CVDs. Risk factors discovered to be significantly associated with plasma proteins include obesity (BMI), smoking behavior, blood lipids (triglycerides), and glucose homeostasis (hemoglobin A1c) (Table S16). Several proteins demonstrated robust mediation effects across multiple outcomes. Insulin-like growth factor binding protein 4 (IGFBP4) and GDF15 showed consistent mediation effects across 9 different CVD outcomes, with median proportion mediated of 27.7% and 25.7%, respectively (Fig. 5B). These proteins mediated the effects of multiple modifiable factors, including BMI, smoking status, and lipid profiles, suggesting their roles as molecular intermediaries in lifestyle-related cardiovascular risk. Other key mediators with broad cardiovascular implications included pro-adrenomedullin (ADM) and serine/threonine-protein kinase receptor R3 (ACVRL1) (both mediating 8 outcomes), and cystatin-C (CST3) (9 outcomes). Protease serine 8 (PRSS8) demonstrated extensive mediation effects, linking 5 different modifiable risk factors to 8 cardiovascular outcomes.

Furthermore, our analysis revealed several notable protein mediators (Fig. 5C). WFDC2 emerged as a particularly strong mediator of smoking-related cardiovascular risks, accounting for 91.6% and 76.9% of the association between smoking and incident heart failure and atrial fibrillation, respectively. Lysosome-associated membrane glycoprotein 3 (LAMP3) and alkaline phosphatase, placental type (ALPP) showed strong mediation effects (> 69%) for smoking-related pulmonary embolism risk. In metabolic pathways, lactadherin (MFGE8) mediated 66.0% of the association between triglycerides and coronary artery disease, Follistatin-related protein 3 (FSTL3) and Collagen alpha-3(VI) chain (COL6A3) were identified as key mediators (> 60%) for BMI-related deep vein thrombosis. LEP, primarily mediating BMI-related effects, exhibited a substantial proportion mediated (50.7%) in coronary artery disease, and also mediated triglyceride effects (17.0%), reinforcing the importance of weight management and lipid control in cardiovascular prevention. The broad network of protein mediators identified here provides molecular evidence supporting the benefits of comprehensive lifestyle interventions in cardiovascular disease prevention (Khera et al., 2016).

### Polygenic risk score reflected the role of genetic risk factors in incident CVDs

**Figure 6.**
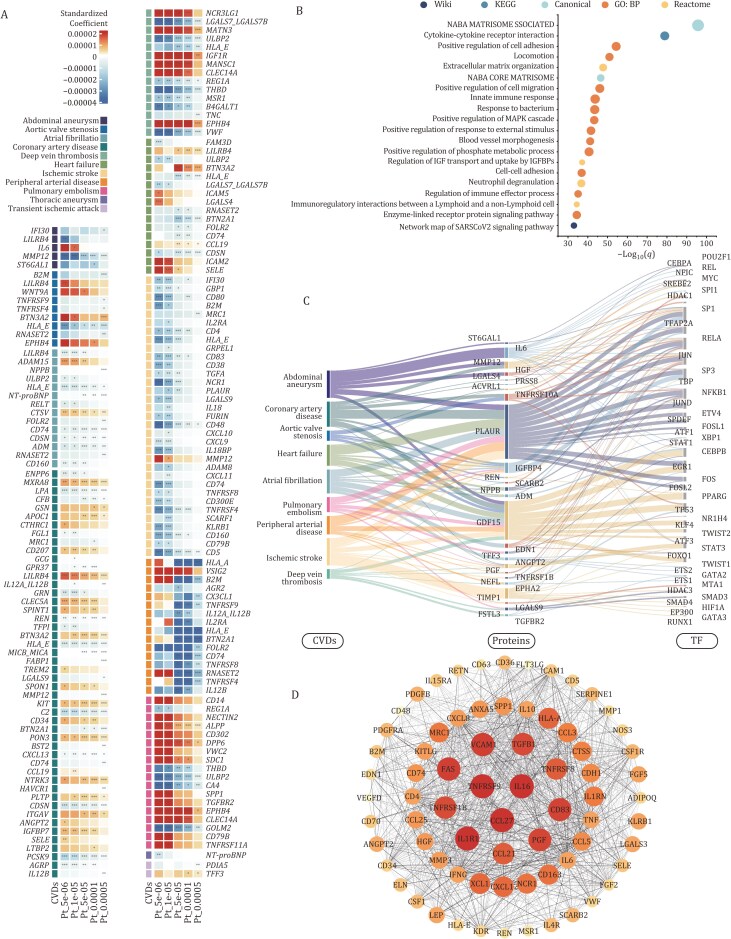
The associations of CVD-associated proteins with genetic factors and functional enrichment analyses. (A) The associations of CVD-associated proteins with genetic risk for CVDs were assessed under five *P*-thresholds (pT_5e-06, pT_1e-05, pT_5e-05, pT_1e-04, pT_5e-04). Linear regression models, adjusted for age, sex, ethnicity, TDI, blood collection season, time between protein measurement and blood sampling, participant-reported fasting time, systolic blood pressure, BMI, smoking status, alcohol intake, and top ten genetic principal components, were utilized. Statistical significance was determined based on two-sided *P*-values, and to address multiple testing, Bonferroni correction was applied (*P* < 0.05/number of CVD-associated proteins). (B) The statistical significance level of each item is shown on the x-axis and is represented by −log_10_ of the *q*-value. y-axis shows different items, the bubble size indicates the enrichment count, and the bubble color indicates the different sources. (C) The protein importance is indicated by the color of nodes, with darker colors indicating higher SCORE2s. The width as well as gray value of the edges indicate the combined SCORE2 between adjacent proteins. Thicker edges and darker shades of gray indicate stronger interactions. (D) The enriched transcription factor targets are all linked to the associated proteins, which in turn connected to the associated CVDs. The length of the color bar indicates the number of correlations involved, with longer bars suggesting more correlations.

To assess the potential influence of genetic risk factors on the observed associations between CVD and protein levels, we explored the relationship between genetic risk for each CVD outcome and corresponding protein levels. Polygenic risk scores for 13 CVD outcomes were constructed based on a comprehensive genome-wide study using five representative *P*-value thresholds (pT_5e-06, pT_1e-05, pT_5e-05, pT_1e-04, pT_5e-04). After Bonferroni correction for each outcome, 126 out of 892 CVD-associated proteins showed statistically significant associations (Fig. 6A and Table S17). Among all 482 significant associations, 139 were discovered in one of the polygenic risk score with coronary artery disease, 78 with ischemic stroke, and 64 with deep vein thrombosis, indicating a strong driving force of genetic risk factors in such types of CVD. Meanwhile, genetic risk factors may not play a role in connecting plasma proteins with cardiomyopathy and intracerebral hemorrhage, emphasizing the importance of other factors in mediating these associations.

### Functional enrichment revealed key pathways of CVD-associated proteins

Pathway enrichment analysis by Metascape unveiled the involvement of 892 CVD-associated proteins in several crucial pathways. Notably, these proteins were associated with Naba matrisome-associated pathway (183 proteins; Canonical pathways), innate immune response pathway (115 proteins; GO Biological Processes), response to bacterium pathway (110 proteins; GO Biological Processes), cytokine-cytokine receptor interaction (105 proteins; KEGG pathway), and positive regulation of cell adhesion (104 proteins; GO Biological Processes) (Fig. 6B and Table S18).

To delve into the regulatory landscape, the top 15 proteins associated with each CVD outcome were selected, and their potential transcription factor targets were identified and visualized using the TRRUST database from Metascape (Fig. 6C and Table S19). A total of 46 transcription factors (TFs) were identified as key regulators of these genes. Among them, well-known factors such as nuclear factor NF-kappa-B p105 subunit (NFKB1), RELA proto-oncogene, NF-κB subunit (RELA), and Sp1 transcription factor (SP1) were recovered, demonstrating the robustness of the predictive model. Importantly, the model also highlighted a set of less-characterized TFs—including JunD proto-oncogene, AP-1 transcription factor subunit (JUND), SAM pointed domain containing ETS transcription factor (SPDEF), ETV4 transcription factor (ETV4), and nuclear factor I C (NFIC)—that have been relatively understudied in the context of cardiovascular disease. These findings suggest novel regulatory mechanisms underlying disease-relevant processes such as vascular remodeling, immune activation, and cellular stress responses.

Furthermore, a protein-protein interaction (PPI) analysis using STRING revealed intricate networks among CVD-associated proteins, yielding a significant network composed of 73 nodes and 2,430 edges. The importance scores, generated by the maximum neighbor component algorithm in cytoHubba, identified interleukin 16 (IL16) and C-C motif chemokine ligand 27 (CCL27) as key cytokines with the highest importance score, underscoring their crucial roles in regulating inflammatory processes (Fig. 6D and Table S20). Two additional networks, encompassing 35 and 50 nodes, further underscore the significance of cytokines in the context of incident CVDs. Notable proteins within these 2 networks included cytokines, such as interleukin 15 (IL15), C-C motif chemokine ligand 4 (CCL4), and so on. Network analysis revealed a highly interconnected protein-protein interaction network centered around inflammatory mediators, with tumor necrosis factor (TNF), interleukin 6 (IL6), and C-C motif chemokine ligand 2 (CCL2) forming major hub nodes that exhibited the strongest connectivity patterns among CVD-associated proteins (Fig. S5, Tables S21 and S22).

## Discussion and conclusion

Our comprehensive analysis of plasma proteome via the UK Biobank database illustrates a multitude of their associations with diverse CVD subtypes. Primarily, Cox proportional hazard models revealed a total of 892 and 784 plasma proteins predominantly linked to 14 subtypes of incident CVDs and mortality, respectively, complemented by associations with CMR metrics via linear regression. Secondly, our developed model on 257 selected proteins demonstrated strong predictive power across 11 CVD outcomes. MR analysis identified 225 proteins causally linked to cardiovascular diseases, with drug target enrichment analysis validating their therapeutic relevance through existing medications and suggesting opportunities for drug repurposing. Moreover, we discovered that the majority of protein-CVD associations are influenced by modifiable risk factors, especially smoking and BMI. Various functional enrichment analyses illustrated the potential transcription factors targets regulating CVDs and emphasized the enriched pathway highly correlated to incident CVDs.

Our Cox model results revealed protein signatures with distinctive cardiovascular risk profiles, proteins, such as NT-proBNP, EDA2R, and GDF15 exhibited a remarkable breadth of associations with 10 CVD outcomes, emphasizing their potential as crucial risk factors for CVD as previously reported (di Candia et al., 2021; Bayes-Genis et al., 2023; Cai et al., 2021; Wang et al., 2020a). The absence of a significant association for subarachnoid hemorrhage suggests distinct underlying mechanisms compared to other CVDs. This may be related to the fact that subarachnoid hemorrhage is specific to bleeding in the subarachnoid space of the brain (Lawton and Vates, 2017), which could be further explored in future research.

While a recent study reported the link of cardiac imaging phenotypes with some risk factors, including lifestyle and early-life factors (Bai et al., 2020), our study stands out as the first to identify a multitude of unique plasma proteins significantly associated with 17 cardiovascular phenotypes in a large cohort. Moreover, the opposing effects observed on the broadly connected proteins underscore the presence of sophisticated homeostatic mechanisms regulating cardiac structure. Notably, chamber-specific patterns of protein associations highlight distinct molecular pathways regulating different cardiac compartments. The negative correlation between LEP and ventricular volumes suggests a protective role against adverse cardiac remodeling (Zhao et al., 2021), consistent with recent experimental evidence linking leptin signaling to cardiac structure maintenance. The associations of FABP4 with right ventricular volumes extend previous observations of its role in left ventricular remodeling, indicating a broader function in cardiac structure regulation (Baessler et al., 2014). These findings suggest that the proteomic landscape mirrors the unique regulatory requirements of individual cardiac chambers. Among the proteins identified, LCAT revealed distinctive associations with cardiac metrics, suggesting a nuanced role in cardiac remodeling. Notably, LCAT exhibited negative correlations with cardiac structural parameters and a positive association with atrial function, indicating its potential to modulate cardiac chamber dynamics and contractility. Such observations indicate a dual regulatory mechanism of LCAT in cardiac adaptation, positioning it as a potential biomarker and therapeutic target for cardiovascular diseases (Lin et al., 2024).

Machine learning techniques have successfully identified proteins with predicting capabilities for multiple incident CVDs. These findings were consistent with the association analyses between these proteins and different CVDs, strengthening their potential as reliable biomarkers. For comparison purposes, we incorporated the CVD risk scale of SCORE2, which has been validated in UK Biobank and numerous studies (Steinfeldt et al., 2022; You et al., 2023). Except for subarachnoid hemorrhage, which might be influenced by various sudden factors, the selected subset of proteins showed significant additive values on top of SCORE2 in all the other CVD outcomes. Further, proteins alone also outperformed the established risk scale across 11 out of 14 outcomes, emphasizing the complementary role of plasma proteins in enhancing the precision and accuracy of predicting CVD risk beyond traditional risk factors. Such prediction models further demonstrated the strong diagnostic ability of plasma proteome in forecasting incident CVDs, offering a new avenue for early disease detection and risk stratification.

Drug target enrichment analysis not only validate our MR-identified proteins (such as PCSK9, F10, LPA, VEGFA) as legitimate therapeutic targets but also highlights potential opportunities for drug repurposing across different cardiovascular conditions. Intriguingly, many of these CVD-associated proteins showed therapeutic applications beyond cardiovascular diseases. Notable examples include significant enrichment in ophthalmological conditions (H30–H36), where VEGFA inhibitors are used, and in metabolic disorders targeted by PCSK9 inhibitors. The identification of these proteins as therapeutic targets across multiple diseases, particularly inflammatory mediators like TNF and IL6, underscores their causal roles in CVD pathways and reinforces the potential for innovative drug repurposing strategies.

Our investigation also revealed common modifiable risk factors contributing to CVD multimorbidity, including smoking, BMI, blood triglyceride and glucose levels, mediated by plasma proteins. These shared risk factors are significant contributors to premature deaths from CVDs, which have been recognized as essential metrics for cardiovascular health (Bays et al., 2022; Hamad et al., 2022; Wang et al., 2023). Our findings emphasize their central role in the potential prevention and management of multimorbidity associated with incident CVDs. Intriguingly, smoking status emerges as a predominant risk factor for abdominal aneurysm, heart failure, and pulmonary embolism, while BMI exhibits specificity for deep vein thrombosis and aortic valve stenosis. Significant mediator proteins, including TNFRSF4, IGFBP7, MMP12, and LTBP2, suggest their roles in mediating the relationship of CVDs with the corresponding risk factors. For instance, MMP12 showed a positive correlation with BMI (Grzechocinska et al., 2019) and was proposed to be a potential biomarker to predict ischemic stroke in patients with obesity (Wang et al., 2020b). These findings emphasize the importance of targeted interventions addressing these modifiable risk factors to mitigate the burden of CVD multimorbidity. While MR analyses suggest causal relationships between certain proteins and CVDs, these findings should be interpreted cautiously, considering the underlying assumptions of genetic instruments. The biological mechanisms linking these proteins to CVD risk likely involve inflammation and endothelial function pathways, as exemplified by MMP12’s role in vascular remodeling (Holm Nielsen et al., 2020). Although residual confounding from lifestyle factors such as diet and physical activity cannot be excluded, our models adjusted for BMI, which is more objectively measured and has shown stronger and more consistent associations with circulating proteins than self-reported lifestyle variables (Trijsburg et al., 2017; Zaghlool et al., 2021). Nonetheless, future work incorporating detailed dietary and physical activity assessments may help further minimize confounding.

Consistent with prior studies, our model validated several established TFs involved in cardiovascular biology, underscoring the model’s accuracy and strength in capturing disease-relevant regulatory mechanisms. NFKB1 and RELA, components of the NF-κB complex, regulate inflammatory signaling (Liu et al., 2017; Zhang et al., 2021) and have been linked to endothelial dysfunction (Donato et al., 2018), cardiac hypertrophy, and fibrosis (Casper, 2023; Hong and Zhang, 2022; Ijaz et al., 2017). SP1 contributes to atherosclerosis by modulating endothelial and smooth muscle cell function (Jiang et al., 2022; Li et al., 2019). Critically, the model also identified several TFs that have received limited attention in cardiovascular research, such as JUND, SPDEF, ETV4, and NFIC. These TFs were predicted to regulate key disease-associated proteins such as PLAUR, GDF15, and IL6, suggesting novel regulatory mechanisms potentially contributing to vascular inflammation, extracellular matrix remodeling, and immune activation. Several of these TFs have established roles in other biological contexts, but their roles in the cardiovascular system remain largely underexplored. For instance, JUND has been linked to macrophage regulation and oncogenesis (Tai et al., 2020; Wang et al., 2014); SPDEF and ETV4 have been studied in epithelial differentiation and cancer progression (Li et al., 2023; Rodriguez et al., 2020); and NFIC, a member of the Nuclear Factor I family, was only recently implicated in ischemic cardiomyopathy (Ye et al., 2022). Their identification highlights their significant potential as therapeutic targets for CVDs and underappreciated regulatory pathways that may contribute to disease pathogenesis, warranting further investigation.

Pathway enrichment and PPI analyses demonstrated that the majority of CVD-associated proteins are involved in the matrisome-associated pathway, crucial for mesenchymal transition and ECM formation (Naba et al., 2012). This pathway was also enriched in human pluripotent stem-cell-derived cardiomyocytes for patient-specific disease modeling (Bayne et al., 2021), further indicating its significance in CVD pathophysiology. In addition to matrisome-associated pathways, our analysis highlighted other key pathways, including the innate immune response, cytokine-cytokine receptor interaction, and positive regulation of cell adhesion. These findings suggest the critical roles of cytokines, particularly interleukin-6 (IL-6), in the progression of CVDs.

Several highlighted proteins above have well-established roles in cardiovascular physiology. Extensive literature has delineated the physiological functions of NT-proBNP, a critical marker for cardiac stress (Bayes-Genis et al., 2023; Murakami et al., 2020), and its precursor NPPB, which is predominantly synthesized by the heart ventricle and serves as a vasodilator that facilitates the blood vessel relaxation (Guan et al., 2020; Wang et al., 2020a). Beyond its established role as a cardiac stress biomarker, NT-proBNP may play a more fundamental role in chamber-specific remodeling than previously recognized (Volpe et al., 2014), potentially participating in the regulation of atrial architecture through local tissue homeostasis pathways. It is worth noting that previous research has investigated the potential of GDF15 in the diagnostics and prognostics of CVDs (di Candia et al., 2021; Negishi et al., 2021; Wang et al., 2020c). However, GDF15 has been recognized as a nonspecific marker, reflecting broader biological processes, including aging, inflammation, and metabolism (di Candia et al., 2021; Breit et al., 2021). Recent studies have also linked the elevated levels of plasma GDF15 to various health conditions, such as obesity (Mastrobattista et al., 2023), diabetes (Hung et al., 2022), cognitive frailty, and depression (Baksh et al., 2023; Kochlik et al., 2024; Mastrobattista et al., 2023), indicating its potential role as a biomarker for systemic health and disease states. In contrast, fewer studies reported the direct role of LTBP2 and EDA2R in CVDs. LTBP2, primarily associated with the ECM, plays a critical role in regulating transforming growth factor-beta (TGF-β), the signaling of which is crucial for vascular development and maintenance. While LTBP2 has been mainly recognized as a prognostic biomarker for idiopathic pulmonary fibrosis (Enomoto et al., 2018), a recent multi-omics study has reported the relationship of plasma LTBP2 with right ventricular dysfunction in human pulmonary arterial hypertension (Boucherat et al., 2022). EDA2R, a receptor for ectodysplasin A2, is a member of the tumor necrosis factor family. It is involved in the development of ectodermal structures and participates in cell signaling pathways related to apoptosis and immune responses (de Vries et al., 2017; Kwack et al., 2019), which are key processes in the development and progression of various CVDs, including myocardial infarction and heart failure. Further research is warranted to elucidate the specific contributions of LTBP2 and EDA2R to different types of incident CVDs.

This study boasts several notable strengths. Firstly, our extended follow-up duration addresses a significant gap in the existing literature, providing a comprehensive longitudinal perspective in an underexplored domain. Moreover, compared to previous studies, our analytical approach encompasses a broader spectrum of CVD outcomes and cardiac structure and function metrics, offering a more comprehensive understanding of the molecular landscape of the disease mechanisms. In contrast to conventional research that often focuses on one or a few types of CVDs at isolated endpoints, our methodology tracks the progression of this complex interplay over time and provides a robust machine learning-based model for disease prediction. Recognizing that incident CVDs result from a multifaceted interplay of genetic, environmental, and biochemical factors, our study integrates these dimensions as well, revealing intricate profiles across diverse populations.

Despite the strengths of this study, several limitations warrant consideration. First, the reliance on the UK Biobank cohort, predominantly comprising Caucasians, may restrict the generalizability of our findings to populations with different ethnic backgrounds. Replication in more ancestrally diverse cohorts is essential for broader applicability. Second, potential horizontal pleiotropy in our MR analysis might affect the robustness of causal inference. Third, although we identified some key proteins and enriched pathways associated with cardiac structure and function, experimental validation is necessary to confirm their biological relevance. Proteins such as LCAT and SEZ6L, which showed strong and chamber-specific associations, represent promising candidates for follow-up studies in cellular or animal models. Together, these efforts will help better translate statistical associations into mechanistic insights and potential clinical applications.

For the first time, we present a comprehensive and systematic exploration of the complicated interrelationships among plasma proteins, genetic factors, risk factors, cardiac imaging metrics, and incident CVDs. Collectively, these findings illuminate the intricate interrelation between the proteome and diverse cardiovascular conditions, offering new insights into the molecular underpinnings of cardiac structure and function and unveiling pathways that may drive the pathogenesis of incident CVDs. To conclude, this study advances our understanding of cardiovascular disease risks, facilitates early-stage disease prediction, and provides valuable biological insights for potential interventions. These findings pave the way for promising developments in future prediction models, diagnostic tools, and preventive strategies for cardiovascular diseases.

## Supplementary Material

pwaf072_Supplementary_Table_1

pwaf072_Supplementary_Figure_1

## Data Availability

UK Biobank data are publicly available to bona fide researchers upon application UKBiobankiobank.ac.uk/using-the-resource/. All data used in this study were accessed from the UK Biobank under application number 19542.

## Code availability

All software used in this study is publicly available. Software and analytical methods used in data analyses include: Cox proportional hazard modeling: the R packages survival v3.5.5. Mendelian randomization analyses were performed using R package TwoSampleMR (v0.5.8). Plink (v2.0) software was available through their website. PRSice (v1.25) software was available through their website. Pathway and up-regulator enrichment analysis was performed using Metascape and phenotype enrichment analysis was performed using data from the Mouse Genome Informatics platform (MGI, 542). STRING was available through their website. Cytoscape software (v3.10.0) was available through their website. Mediation analysis: the R packages CMAverse v0.1.0. R v.4.3.2 was used in general for statistical analyses and plotting.
