# Supplementary material for: Systematic analyses uncover plasma proteins linked to incident cardiovascular diseases
Source: Protein Cell. 2025 Aug 6;17(3):231–47. doi: 10.1093/procel/pwaf072 (PMC12987571; doi:10.1093/procel/pwaf072)
Supplement: pwaf072_Supplementary_Figure_1 [file pwaf072_supplementary_figure_1.pdf]

Supplementary Notes

Methods

Study participants

The UK Biobank, a prospective population cohort, recruited more than half a million healthy adults aged 40-69 years from 21 assessment centers throughout Scotland, Wales, and England between 2006 and 2010. Participant data include extensive baseline information, along with follow-up outcomes derived from periodic updates of biochemical and clinical measurements. Ethical approval of the UK Biobank study was granted by the NHS North West Multicenter Research Ethics Committee, and informed consent was obtained from all participants during recruitment.

Plasma proteomics

In the UK Biobank Pharma Proteomics Project, the proteome of plasma samples collected from 54,306 participants in UK Biobank was quantified by the Olink Explore 3072 platform consisting of 8 panels of 384 assays. The measures of 2,923 unique proteins, categorized into 4 Olink panels (Cardiometabolic, Inflammation, Neurology, and Oncology), underwent rigorous quality control. The normalized protein eXpression (NPX) values were generated by the manufacturer via protein concentration normalization and extensive quality control measures (Sun et al., 2023). After excluding proteins with a missing rate of more than 30%, 2,920 distinct proteins were included (**Table S23**).

Endpoints

We included a broad range of CVDs, including ischaemic stroke, hemorrhagic stroke, transient ischaemic attack, pulmonary embolism, deep vein thrombosis, arterial hypertension, atrial fibrillation, chronic ischaemic heart disease, coronary artery disease, myocardial infarction, cardiomyopathy, heart failure, and peripheral artery disease. The diagnosis of CVD and mortality cases were obtained from the first occurrence, hospital inpatient, death register and algorithmically-defined outcomes. Further detailed procedures can be accessed on UK Biobank's official website: https://www.ukbiobank.ac.uk/wp-content/uploads/2011/11/UKBiobank-Protocol.pdf. All outcome events were identified utilizing the International Classification of Diseases-10th revision (ICD-10) codes, and the relevant ICD-9 coding system was considered as well. A full list of phenotype definitions is shown in **Table S24**. Follow-up duration was calculated from participating dates in the assessment center to the first-occurred date of incident CVD diagnosis, censoring or death.

CMR imaging data

The detailed UK Biobank CMR protocol has been reported by Petersen, S. E. et al previously (Petersen et al., 2016). Briefly, a clinical wide-bore 1.5-T scanner (MAGNETOM Aera, Syngo PlatformVD13A [Siemens Healthcare]) was applied to perform CMR imaging. Long-axis 4-chamber imaging was utilized for LA and RA segmentation, while the RA, myocardium and LV segmentation was acquired on a complete short-axis stack at an average rate of 1 slice per breath hold. Following CMR indices were considered in our current analyses: LA ejection fraction (LAEF), LA stroke volume (LASV), LA maximum volume (LAV max), LA minimum volume (LAV min), LV cardiac output (LVCO), LV end-diastolic volume (LVEDV), LV ejection fraction (LVEF), LV end-systolic volume (LVESV), LV

myocardial mass (LVM), LV stroke volume (LVSV), RA ejection fraction (RAEF), RV stroke volume (RASV), RA maximum volume (RAV max), RA minimum volume (RAV min), RV end-diastolic volume (RVEDV), RV end-systolic volume (RVESV), RV stroke volume (RVSV), and global wall thickness for the LV mean myocardial. Detailed measurements were available in previous studies.

Statistical analysis

As for the participants' baseline characteristics, the mean (SD) was used to describe continuous variables while frequency (proportion) for categorical variables. We applied Cox proportional hazard regression models to determine the association between circulating protein levels and the risk of CVD, accompanied by age, sex, ethnicity, Townsend deprivation index, smoking status, alcohol consumption, BMI and systolic blood pressure as covariates. The results were expressed as HR and 95% Cl. Follow-up duration was from baseline to the earliest date of initial diagnosis, hospital admission, death, or loss to follow-up. For incident CVD analysis, we excluded participants with any type of CVDs at baselines. A P-value of less than the threshold after Bonferroni correction ($P < 0.05/2,920$) indicated statistical significance. The Cox proportional hazards models were implemented using the ‘coxph’ function from the R ‘survival’ package (v3.5.5). Multiple linear regression models were executed with imaging phenotype as the outcome and each protein as the exposure. To reduce the impact of pre-existing cardiac conditions on imaging phenotypes, participants with any CVDs at baseline and those without imaging phenotypes were excluded. This resulted in a final sample of 4,287 participants for analysis. In the sensitivity analysis, we further included creatinine, cystatin-C, LDL-cholesterol, HDL-cholesterol, C-reactive protein, hemoglobin,

and HbA1c as covariates in our models. To account for the competing risks when evaluating long-term outcomes, competing risk models were used to evaluate the associations between key proteins and long-term outcomes.

Within the derivation cohort, a union set of 671 proteins, survived after Bonferroni correction (with *P-values* < 0.05) for each CVD outcome, was included into a machine learning pipeline to develop prediction models. This pipeline involved two main steps: protein selection and model development. The proteins were selected based on feature importance ranking using information gain. For each endpoint, top proteins accounts for total 30% of the normalized importance were selected, resulting in a union of 257 proteins across 14 CVD outcomes. We then leveraged the light gradient boosting machine (LGBM) classifiers (Ke et al., 2017) to predict CVD risk, comparing models using three predictor sets: Protein (257 proteins), SCORE2 (an established CVD risk scale)(group and collaboration, 2021), and Protein+SCORE2. Predictive performance was evaluated through ROC analysis and multiple metrics. Models were trained and validated based on an internal data partition into two-third as model derivation set and the remaining as hold-out test set. Notably, the testing set was kept untouched and merely used for model evaluation, which was performed using bootstrap strategies to ensure robustness. DeLong statistics were applied to test the significant difference between paired areas under the curve (AUCs) (DeLong et al., 1988) and net reclassification improvement (NRI) and integrated discrimination improvement (IDI) were calculated to evaluate the additive clinical values of proteins beyond SCORE2.

We conducted a two-sample MR analysis to investigate whether CVD-associated proteins have a causal effect on CVDs. In addition, we conducted a three-variable path

mediation analysis to examine whether circulating protein levels mediated the association between vascular traits and CVDs. Moreover, Enrichment for targets of drugs categorized according to indications which are coded by the ICD10 diagnostic system was carried out utilizing the Genome for Repositioning(Sakaue and Okada, 2019). We performed functional enrichment analysis using Metascape to understand the biology of CVD-associated proteins, including pathways and gene ontology (GO) terms. Phenotype enrichment analysis and transcriptional regulatory network exploration using TRRUST identified upstream regulators of these proteins. Additionally, we conducted PPI network analysis using STRING (https://string-db.org/) and Cytoscape to visualize and rank important protein interactions.

Detailed methods are provided as below.

Covariates

In our present analyses, we adjusted for demographic variables, including age, gender, ethnicity (white/non-white) and socioeconomic status (reflected through the Townsend deprivation index), which were obtained by a touchscreen questionnaire. Potential confounding factors correlated with CVD were also considered as covariates in the current analysis. BMI was calculated via height and weight collected at recruitment (weight/height2). Smoking status was self-reported at baseline and classified as never, former, and current smoker. Alcohol consumption information from the touchscreen questionnaire was categorized as never, former, and current as well. SBP was derived from two blood pressure measurements with manual or automated devices for each individual. In addition, the time between protein measurement and blood sampling (in days), participant-reported fasting time, and blood collection season

(winter/spring [December to May] versus summer/autumn [June to November]) were considered to assess bias in protein detection levels. More specific information on covariates is detailed in **Table S25**. For missing values, continuous variables were interpolated with medians, discrete variables with plurality, Townsend deprivation index with site medians, and ethnicity with white Europeans, while SBP and BMI were interpolated using medians of different gender groups.

Identification of CVD-associated proteins

As for the participants' baseline characteristics, the mean (SD) was used to describe continuous variables while frequency (proportion) for categorical variables. We applied Cox proportional hazard regression models to determine the association between circulating protein levels and the risk of CVD, accompanied by age, sex, ethnicity, Townsend deprivation index, smoking status, alcohol consumption, BMI and systolic blood pressure as covariates. The results were expressed as HR and 95% Cl. Follow-up duration was from baseline to the earliest date of initial diagnosis, hospital admission, death, or loss to follow-up. For incident CVD analysis, we excluded participants with any type of CVDs at baselines. For missing values, continuous variables were interpolated with medians, discrete variables with plurality, Townsend deprivation index with site medians, and ethnicity with white Europeans, while SBP and BMI were interpolated using medians of different gender groups. A p-value of less than the threshold after Bonferroni correction ($P < 0.05/2,920$) indicated statistical significance. The Cox proportional hazards models were implemented using the 'coxph' function from the R 'survival' package (v3.5.5).

Linear analysis

Multiple linear regression models were executed with imaging phenotype as the outcome and each protein as the exposure, adjusting for age, sex, ethnicity, Townsend deprivation index, smoking status, alcohol consumption, BMI, and systolic blood pressure as covariates. To minimize the influence of pre-existing cardiac conditions on imaging phenotypes, participants with any types of CVDs at baseline were excluded. Furthermore, participants without imaging phenotypes were also excluded. Ultimately, 4,287 participants were included in this analysis.

Predictive model development and evaluation

To investigate the predictive capability of proteomics profiles, we leveraged machine learning to establish prediction models for incident CVD events. To avoid overfitting, the analysis partitioned the data into a derivation set (two-thirds individuals) and a hold-out test set (remaining one-third individuals). The protein selection and model development were performed within the derivation set. The test set was kept untouched and used merely for evaluation purpose.

Machine learning models were established for each CVD endpoint, including two steps, protein selection and model establishment. For protein selection, we initially leveraged the union set of proteins (n=671) that survived association analysis p-value < 0.05 after Bonferroni corrections under each CVD outcome. Next, we calculated the protein importance, which was determined based on information gain derived from preliminary trained LGBM classifiers (Ke et al., 2017). The information gain was defined as:

$$IG(S,A) = Entropy(S) - \sum_{v \in Values(A)} \frac{|S_v|}{|S|}$$

where the entropy of the orinal dataset S is:

$$Entropy(S) = -\sum_{i=1}^{c} p_i \, log_2(p_i)$$

Specifically, c represents the number of classes in the target variable and p_i denoted as proportion of instances in class c within S. The weighted entropy after splitting on feature A is:

$$\sum_{v \in Values(A)} \frac{|S_v|}{|S|} \, Entropy(S_v)$$

Specifically, $Values(A)$ is the distinct values of predictor A; $|S_v|$ is the number of instances where predictor A has value v; $|S|$ is the total number of instances in S.

After that, for each CVD outcome, protein importance scores were normalized to have an overall summed score equal to one. We then aggregated top proteins account for at least 30% of overall summed importance under each CVD outcome. The union set of proteins (n=257) selected under 14 CVD endpoints was used to develop prediction models for each CVD endpoint. We employed LGBM to develop models to classify healthy individuals (predicted as 0) or future events (predicted as 1). The LGBM were tuned using 10-fold cross-validation within the derivation set, with key hyperparameters as {*n_estimators*=500; *max_depth*=15; *num_leaves*=10; *subsample*=0.7; *learning_rate*=0.01; *colsample_bytree*=0.7}. The machine learning model development was implemented with the lightgbm library (v3.3.2) in Python (v3.9).

To demonstrate the additive value of plasma proteins on top of established risk scales, we employed three sets of predictors, namely, Protein (pre-selected 257 proteins), SCORE2 (an established CVD risk scale that incorporates individual's age, total cholesterol, HDL cholesterol, systolic blood pressure, prevalent diabetes, and smoking status) (Ke et al., 2017), and Protein+SCORE2 (combination of 257 proteins and SCORE2 scale). Of note, the SCORE2 was recalibrated through isotonic regression in the UKB(Chakravarti, 1989). The established models were deployed in the test set and predicted risks were evaluated using a bootstrap strategy with 1,000 iterations to report the median and 95% confidence intervals of statistics. The predictive performance was assessed via Receiver Operating Characteristic analysis, using discrimination evaluated through the AUC and other metrics, e.g., accuracy, sensitivity, specificity, F1-score and Brier score. DeLong statistics were applied to test the significant difference between paired AUCs (DeLong et al., 1988). In addition, to evaluate the additive clinical value of proteins, we calculated the net reclassification improvement (NRI) and integrated discrimination improvement (IDI) for Protein+SCORE2 model versus SCORE2 alone.

Polygenic risk scoring

Genotype data used for Polygenic Risk Scores (PRS) calculation were available in the UKB cohort. We excluded SNPs with Minor allele frequency < 1%, Hardy–Weinberg equilibrium p-value < 1×10^{-6} and the missingness > 1%. We also selected subjects according to the following parameters: (1) The missingness of variants < 1%; (2) Do not show putative sex chromosome aneuploidy; (3) Do not mark as outliers for heterozygosity and missing rates; (4) Have at most ten putative third-degree relatives. To minimize bias due to sample overlaps, GWAS summary

data in which participants did not consist of UKB individuals was utilized. Details of relevant GWAS studies are available in **Table S26**.

We performed PRS via PRSice (www.PRSice.info), based on p-value clumping of $R^2 > 0.1$ within a 250 kb window. Five representative p-value thresholds were considered in our PRS calculation, including $P < 5\times10^{-6}$, $P < 1\times10^{-5}$, $P < 5\times10^{-5}$, $P < 1\times10^{-4}$ and $P < 5\times10^{-4}$. In addition, each prediction model incorporated the first 10 principal components as covariates.

Mendelian randomization analysis

This study utilized data from the UK Biobank, a large-scale prospective cohort study conducted across multiple centers in the United Kingdom from 2006 to 2010. The study included 53,026 participants who met the eligibility criteria of having complete proteomic data and no history of CVD at baseline. All participants provided written informed consent, and the study was approved by the NHS National Research Ethics Service. To investigate whether CVD-associated proteins exert a causal effect on CVDs, we carried out a two-sample Mendelian randomization analysis with the GWAS summary data displayed in **Table S24** for outcomes, while the relevant summary results for each protein were derived from a previous study (Sun et al., 2023). The MR analysis relied on three key assumptions: (1) genetic variants are strongly associated with protein levels, (2) genetic variants are not associated with confounders, and (3) genetic variants affect outcomes only through their effects on protein levels.

We extracted exposure-associated SNPs with a p-value $< 5.0 \times 10^{-8}$ and clumped these SNPs based on a 1000 kb window with $r^2 > 0.01$ to select independent instrumental variables (IVs), whereas the p threshold was relaxed to 1×10^{-6} in case of insufficient number of IVs (IVs

≤ 2) to ensure the robustness of our MR results. The IVW method was presented as the main tool for predicting potential causal relationships. The MR-Egger and weighted-median methods, meanwhile, provide additional in-depth sensitivity analyses for the robustness of our findings. Effect sizes were reported as odds ratios with 95% confidence intervals per standard deviation increase in protein levels. Multiple testing correction was performed using the Benjamini-Hochberg procedure with a false discovery rate of 5%. Missing data were handled using complete case analysis. All related analyses were conducted using the "TwoSampleMR" R package (v0.5.8).

Mediation analysis

To test whether the association of vascular traits with CVDs was mediated by circulating protein levels, we conducted a three-variable path mediation analysis. Before constructing the risk factor–proteins–diseases triangles, we ensured the significance and direction of the association of proteins-diseases (cox regression, $P < 0.05/2,920$), clinical factor-diseases (linear regression, $P < 0.05$) and clinical factor-proteins (cox regression, $P < 0.05/2,920$). Based on the results, risk factor–proteins–diseases triangles were constructed where all three components showed consistent association directions in the hypothesized path. Causal mediation analyses (VanderWeele, 2016) were then performed for each triangle to explore the potential mediating role of proteins in the relationship between risk factors and incident chronic diseases. Our approach involved two regression models: a linear model to regress the protein on the clinical factor and a Cox proportional hazard model to regress the disease on the clinical risk factor and the protein. Both regressions were adjusted for various covariates, including age, sex, ethnicity, education, employment, TDI, smoking status, alcohol intake, BMI, and SBP. This allowed us

to estimate three key factors: the total effect, total natural indirect effect (TNIE), and total natural direct effect (TNDE). To gauge the extent of mediation, we calculated the proportion of the mediating effect, expressed as the mediator (TNIE/[TNDE+TNIE]). To ensure robustness, we employed bootstrapping with 1,000 replications to obtain accurate 95% confidence intervals for our estimates. The mediation analysis was conducted using the 'cmest' function from the R 'CMAverse' package (v0.1.0).

Drug ability assessment

Enrichment for targets of drugs categorized according to indications which are coded by the ICD10 diagnostic system was carried out utilizing the Genome for Repositioning (Sakaue and Okada, 2019). DrugBank (https://www.drugbank.ca/) and Therapeutic Target Database (http://bidd.nus.edu.sg/BIDD-Databases/TTD/TTD.asp) are freely available databases with regularly updated and experimentally validated data on drug targets, in which genes with $q < 0.05$ were extracted to enrich for drug target genes.

Functional enrichment analysis

We utilized Metascape (Zhou et al., 2019), a powerful tool providing gene function annotation, to achieve a thorough comprehension of the biology of CVD-associated proteins. The key target proteins were imported into Metascape to examine various functional enrichment, including KEGG pathways, GO terms, Canonical pathways, Reactome pathways and WikiPathways.

We performed phenotype enrichment analysis for more biological significance of CVD-associated proteins. We obtained phenotypically annotated genes encoding CVD-associated proteins through the Mouse Genome Information platform (http://www.informatics.jax.org/),

which delivers access to biological, genomic and genetic data on experimentally studied mice. Fisher's exact test was applied to determine errors in the ratio of phenotype-associated genes from the gene set and background gene set.

In addition, we used TRRUST (Han et al., 2018) to visualize human transcriptional regulatory networks, and explored the upstream regulators of our identified proteins together with Metaspace. TRRUST contains 8,444 human-derived and 6,552 rat-derived transcription factor (TF) target interactions. Based on this analysis, we identified TFs that may control the expression of CVD-associated proteins.

PPI network and importance ranking

STRING (https://string-db.org/), a functional protein association tool, was applied to conduct a thorough PPI network analysis. Subsequently, Cytoscape software (Shannon et al., 2003), in which the MCODE and cytoHubba plugin served to screen modules and identify hub genes, was employed to generate a visual depiction. We then used the MCODE algorithm to identify regions of this PPI network where proteins were densely connected.

References

CHAKRAVARTI, N. 1989. Isotonic median regression: a linear programming approach. *Math. Oper. Res.,* 14**,** 303–308.

DELONG, E. R., DELONG, D. M. & CLARKE-PEARSON, D. L. 1988. Comparing the areas under two or more correlated receiver operating characteristic curves: a nonparametric approach. *Biometrics,* 44**,** 837-45.

GROUP, S. W. & COLLABORATION, E. S. C. C. R. 2021. SCORE2 risk prediction algorithms: new models to estimate 10-year risk of cardiovascular disease in Europe. *Eur Heart J,* 42**,** 2439-2454.

HAN, H., CHO, J. W., LEE, S., YUN, A., KIM, H., BAE, D., YANG, S., KIM, C. Y., LEE, M., KIM, E., LEE, S., KANG, B., JEONG, D., KIM, Y., JEON, H. N., JUNG, H., NAM, S., CHUNG, M., KIM, J. H. & LEE, I. 2018. TRRUST v2: an expanded reference database of human and mouse transcriptional regulatory interactions. *Nucleic Acids Res,* 46**,** D380-D386.

KE, G., MENG, Q., FINLEY, T., WANG, T., CHEN, W., MA, W., YE, Q. & LIU, T.-Y. 2017. LightGBM: a highly efficient gradient boosting decision tree. *Proceedings of the 31st International Conference on Neural Information Processing Systems.* Long Beach, California, USA: Curran Associates Inc.

PETERSEN, S. E., MATTHEWS, P. M., FRANCIS, J. M., ROBSON, M. D., ZEMRAK, F., BOUBERTAKH, R., YOUNG, A. A., HUDSON, S., WEALE, P., GARRATT, S., COLLINS, R., PIECHNIK, S. & NEUBAUER, S. 2016. UK Biobank's cardiovascular magnetic resonance protocol. *J Cardiovasc Magn Reson,* 18**,** 8.

SAKAUE, S. & OKADA, Y. 2019. GREP: genome for REPositioning drugs. *Bioinformatics,* 35**,** 3821-3823.

SHANNON, P., MARKIEL, A., OZIER, O., BALIGA, N. S., WANG, J. T., RAMAGE, D., AMIN, N., SCHWIKOWSKI, B. & IDEKER, T. 2003. Cytoscape: a software environment for integrated models of biomolecular interaction networks. *Genome Res,* 13**,** 2498-504.

SUN, B. B., CHIOU, J., TRAYLOR, M., BENNER, C., HSU, Y. H., RICHARDSON, T. G., SURENDRAN, P., MAHAJAN, A., ROBINS, C., VASQUEZ-GRINNELL, S. G., HOU, L., KVIKSTAD, E. M., BURREN, O. S., DAVITTE, J., FERBER, K. L., GILLIES, C. E., HEDMAN, A. K., HU, S., LIN, T., MIKKILINENI, R., PENDERGRASS, R. K., PICKERING, C., PRINS, B., BAIRD, D., CHEN, C. Y., WARD, L. D., DEATON, A. M., WELSH, S., WILLIS, C. M., LEHNER, N., ARNOLD, M., WORHEIDE, M. A., SUHRE, K., KASTENMULLER, G., SETHI, A., CULE, M., RAJ, A., ALNYLAM HUMAN, G., ASTRAZENECA GENOMICS, I., BIOGEN BIOBANK, T., BRISTOL MYERS, S., GENENTECH HUMAN, G., GLAXOSMITHKLINE GENOMIC, S., PFIZER INTEGRATIVE, B., POPULATION ANALYTICS OF JANSSEN DATA, S., REGENERON GENETICS, C., BURKITT-GRAY, L., MELAMUD, E., BLACK, M. H., FAUMAN, E. B., HOWSON, J. M. M., KANG, H. M., MCCARTHY, M. I., NIOI, P., PETROVSKI, S., SCOTT, R. A., SMITH, E. N., SZALMA, S., WATERWORTH, D. M., MITNAUL, L. J., SZUSTAKOWSKI, J. D., GIBSON, B. W., MILLER, M. R. &

WHELAN, C. D. 2023. Plasma proteomic associations with genetics and health in the UK Biobank. *Nature,* 622**,** 329-338.

VANDERWEELE, T. J. 2016. Mediation Analysis: A Practitioner's Guide. *Annu Rev Public Health,* 37**,** 17-32.

ZHOU, Y., ZHOU, B., PACHE, L., CHANG, M., KHODABAKHSHI, A. H., TANASEICHUK, O., BENNER, C. & CHANDA, S. K. 2019. Metascape provides a biologist-oriented resource for the analysis of systems-level datasets. *Nat Commun,* 10**,** 1523.

Supplementary Figures

Systematic analyses uncover plasma proteins linked to incident cardiovascular diseases

Figure S1 Summary of event distribution during follow-up.

Figure S2 Manhattan plot summarizing results from Cox proportional hazard models.

Figure S3 Brick plot showing the ranking of proteins based on the number of associated incident CVDs (a) and mortality (b).

Figure S4 Summary of sensitivity analysis.

Figure S5 PPI results for CVD-associated proteins.

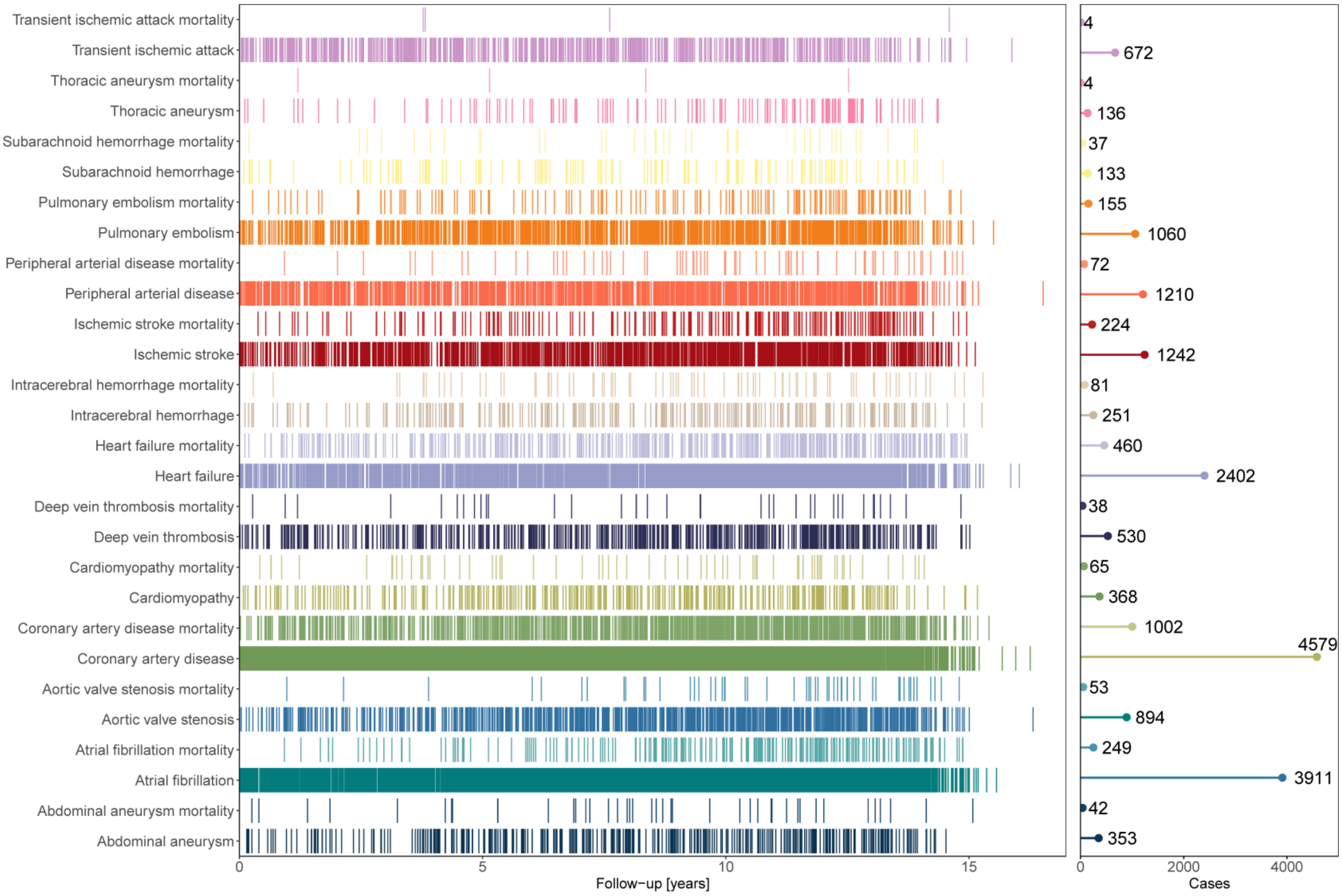

Figure S1 |. Summary of event distribution during follow-up.

Occurrence of events during follow-up. Each line indicates an event. The pin plot on the right gives the total number of cases for each outcome (n = 53,026).

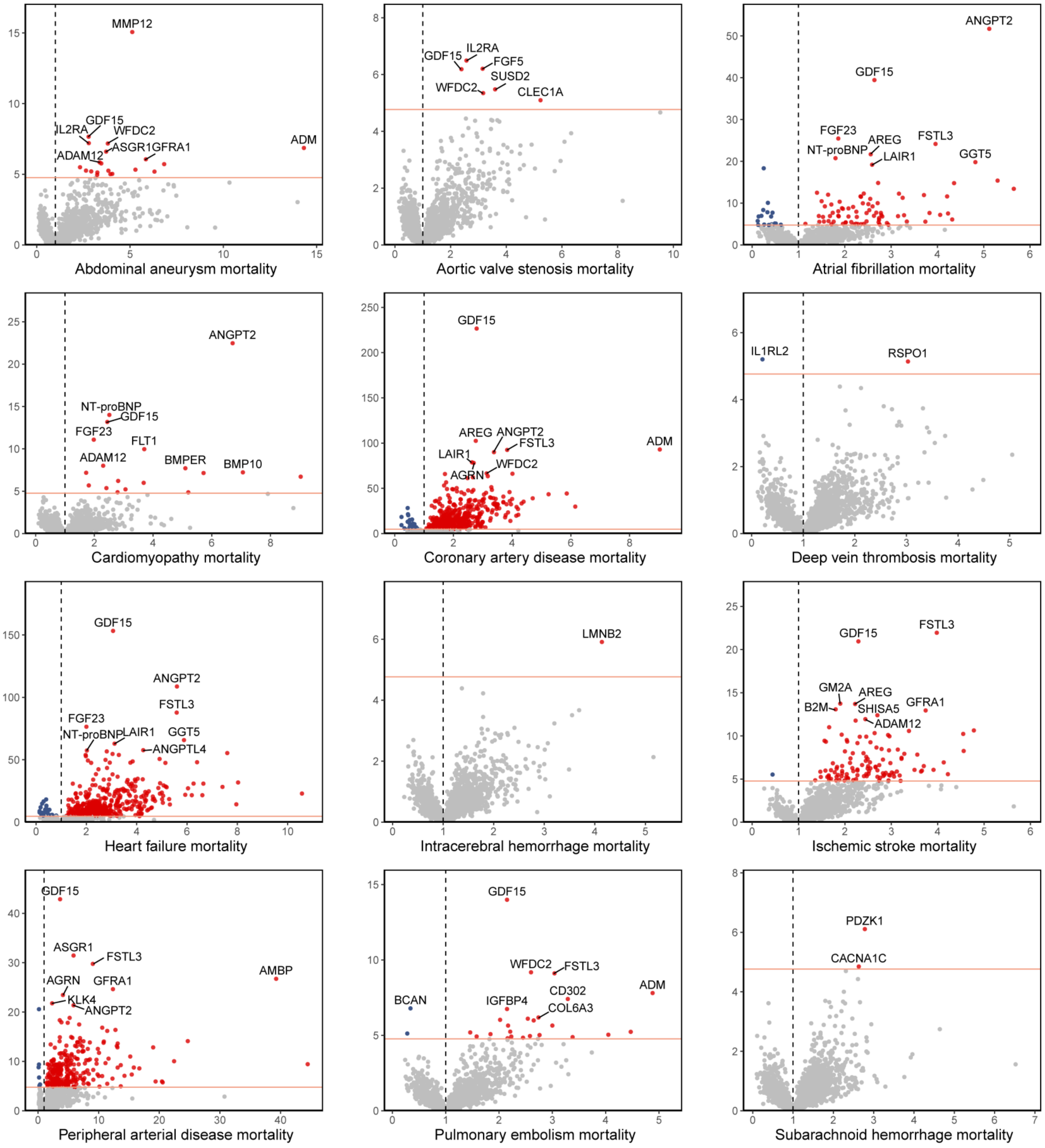

Figure S2 |. Manhattan plot summarizing results from Cox proportional hazard models.

Volcano plots presenting the association of protein expressions with each outcome. The x-axis shows the HR, indicating the strength and direction of the association between protein levels and incident. The y-axis indicates the -log10 of the P value for each association. Proteins with a P value below the threshold determined by the Bonferroni correction (P < 0.05/2,920), are depicted above the black horizontal line. The red dots represent risk proteins, suggesting that they are significantly associated with an enhanced risk of incident CVD, while the blue dots represent protective proteins, indicating that they are significantly associated with a reduced risk of incident CVD. The top 8 proteins with highest $-\log_{10}$ (p-value) for each outcome are marked with their names. The Cox proportional hazard models were adjusted for for age, sex, ethnicity, TDI, blood collection season, time between protein measurement and blood sampling (in days), participant-reported fasting time, systolic blood pressure, BMI, smoking status, and alcohol intake.

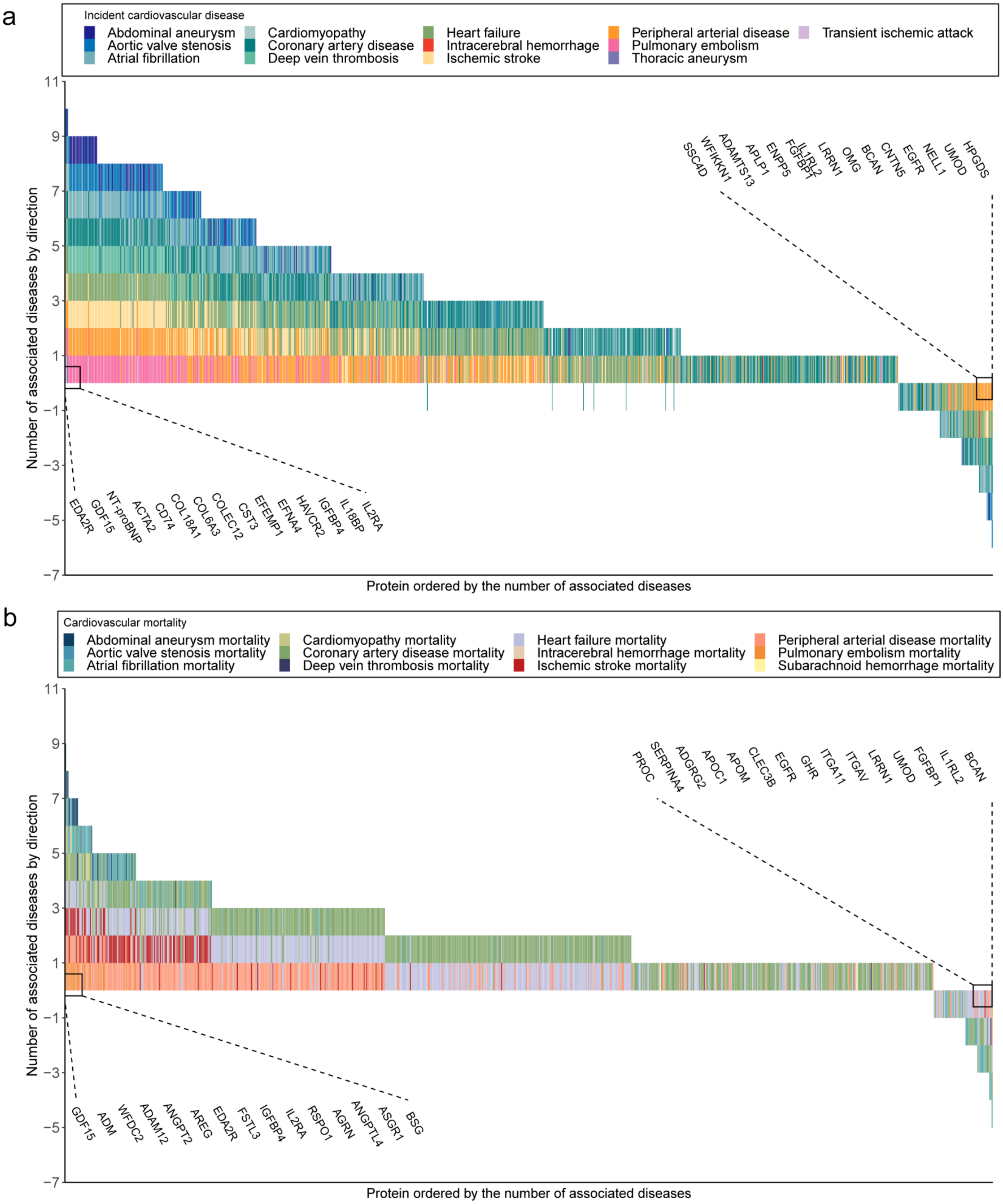

Figure S3 |. Brick plot showing the ranking of proteins based on the number of associated incident CVDs (a) and mortality (b).

The Cox proportional hazard models were adjusted for for age, sex, ethnicity, TDI, blood collection season, time between protein measurement and blood sampling (in days), participant-reported fasting time, systolic blood pressure, BMI, smoking status, and alcohol intake. P < 0.05/2,920 was considered significant accounting for multiple comparisons. The x axis displays the rank of each according to the number of associated proteins, counting inverse associations as negative numbers to ease representation of the results. The y axis counts the number of associated outcomes, whereby positive numbers indicate positive associations and negative numbers indicate inverse associations. The colors of each box indicate the associated outcome.

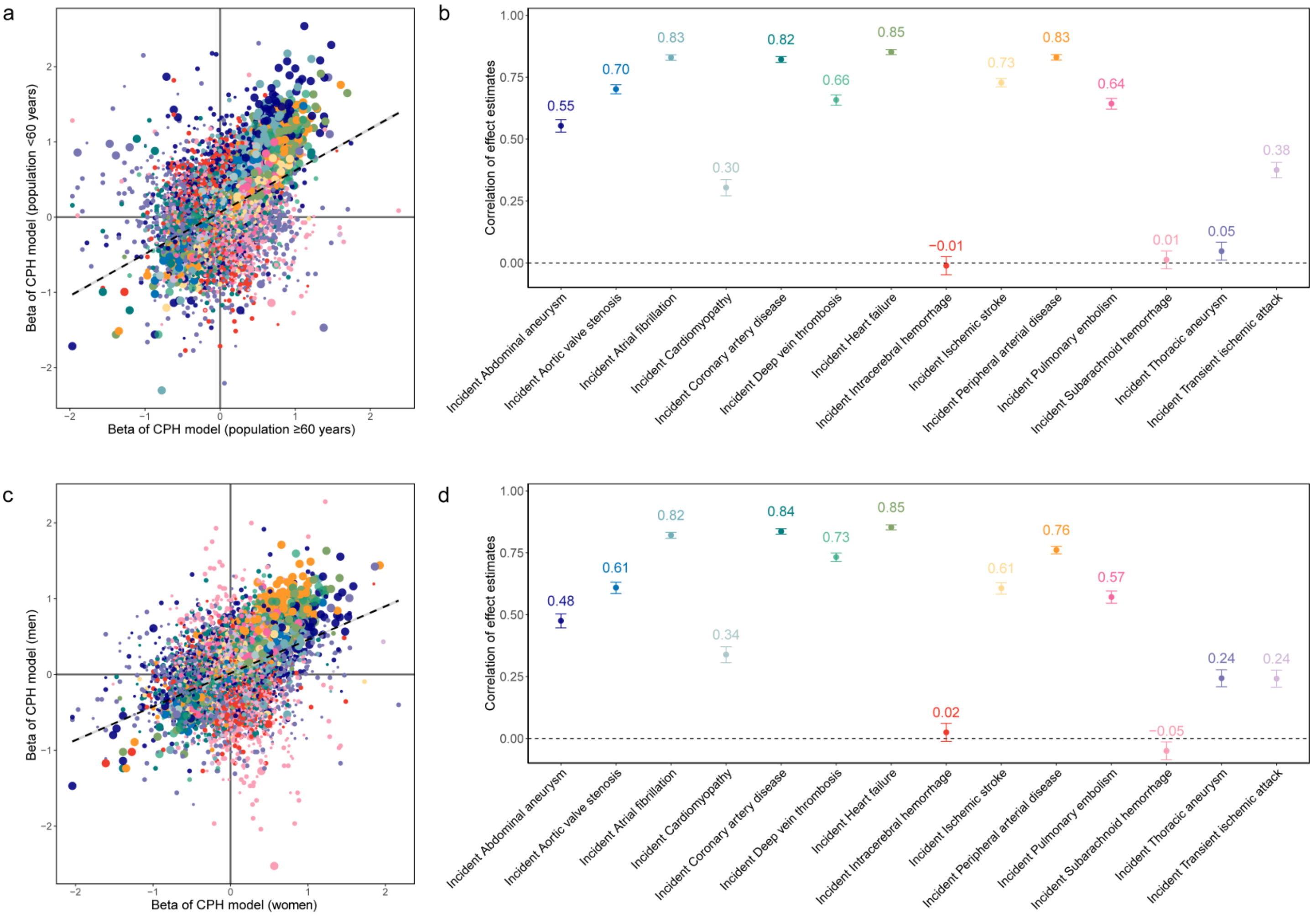

Figure S4 |. Summary of sensitivity analysis.

a, Comparison of effect estimates obtained from Cox proportional hazard models for the different age groups (<60/⩾60 years). **b,** Correlation coefficients, demonstrating the relationships between effect estimates across all proteins for a specific incident outcome between age groups. **c,** Comparison of effect estimates obtained from Cox proportional hazard models between males and females. **d**, Correlation coefficients, demonstrating the relationships between effect estimates across all proteins for a specific incident outcome between sexes. The colors of each dot indicate the associated outcome.

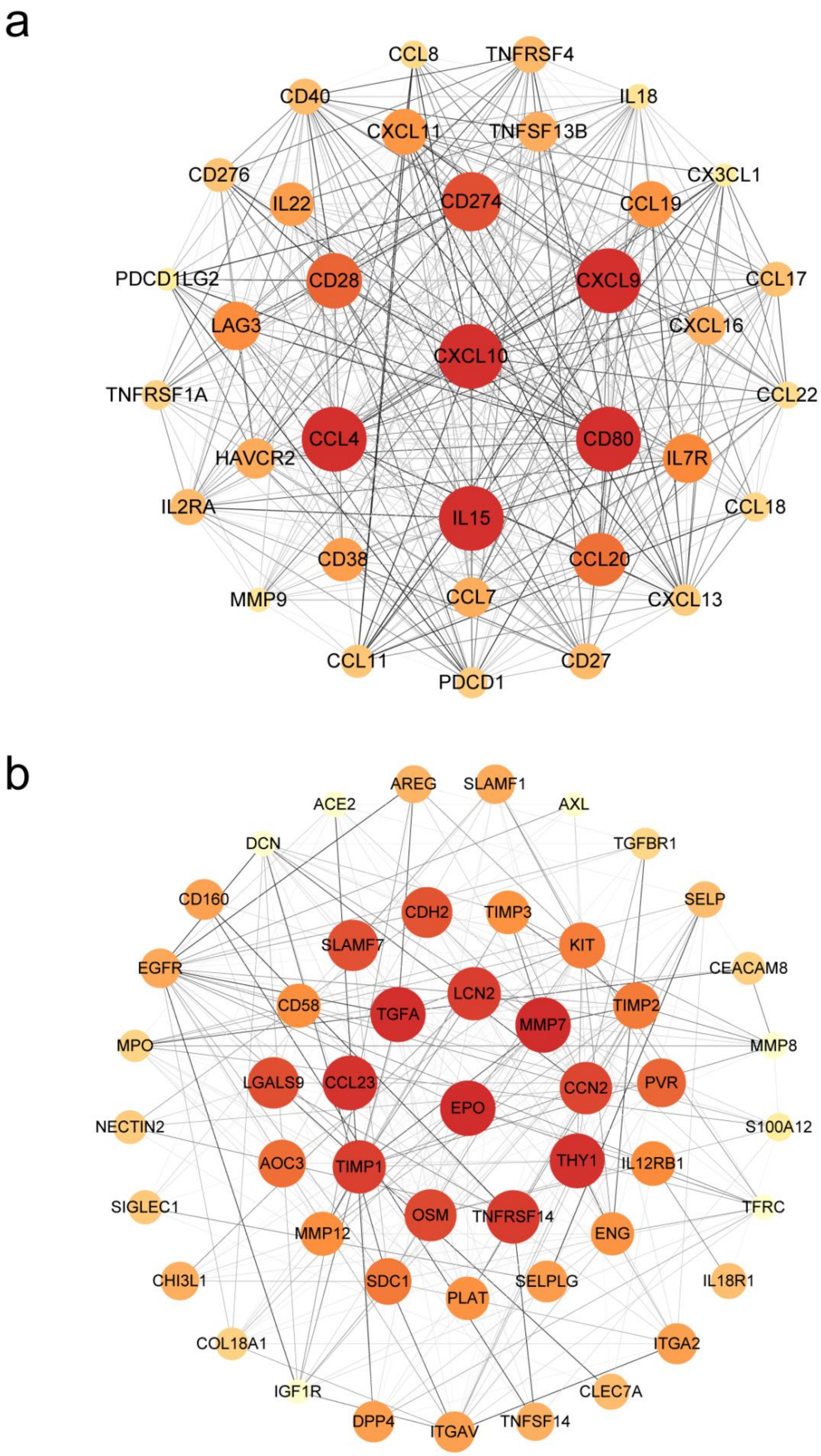

Figure S5 |. PPI results for CVD-associated proteins.

a, The protein importance is indicated by the colour of nodes, with darker colours indicating higher scores. The width as well as grey value of the edges indicate the combined score between adjacent proteins. Thicker edges and darker shades of grey indicate stronger interactions. **b,** Two other notable MCODE modules identified from PPI analysis.
